# Transition metal ion-doped In_2_O_3_ nanocubes: investigation of their photocatalytic degradation activity under sunlight†

**DOI:** 10.1039/d0na00694g

**Published:** 2020-11-19

**Authors:** Velayutham Shanmuganathan, Jayaraj Santhosh Kumar, Raman Pachaiappan, Paramasivam Thangadurai

**Affiliations:** Centre for Nanoscience and Technology, Pondicherry University Kalapet Pondicherry 605014 India thangadurai.p@gmail.com thangaduraip.nst@pondiuni.edu.in; Department of Biotechnology, School of Bioengineering, SRM University Kattankulathur 603 203 Tamilnadu India

## Abstract

The objective of this work was to study the effect of transition metal ion doping (1 wt% of Mn, Fe, Co, Ni, and Cu) in indium oxide (In_2_O_3_) on its photocatalytic activity to degrade organic dyes, which are considered potential environment pollutants. The transition metal ion-doped In_2_O_3_ nanocube photocatalyst was prepared *via* the hydrothermal method. After understanding the thermal behavior of the as-prepared sample (In(OH)_3_), it was calcined at 400 °C for 3 h to obtain In_2_O_3_. The In_2_O_3_ was systematically investigated *via* FESEM, X-ray diffraction, Raman spectroscopy and UV-vis absorption analysis. Microstructure analysis by FESEM showed that the In_2_O_3_ was formed as nanocubes. These nanocubes were formed in a single phase with a cubic crystal structure, while their crystallite size increased from 11 nm to 19 nm when doped with 1 wt% of transition metals, including Mn, Fe, Co, Ni and Cu. The band gap energy for pure In_2_O_3_ was determined to be 3 eV, and that for the metal ion-doped In_2_O_3_ showed a slight decrease to the lowest value of 2.94 eV. The photoluminescence (PL) decay lifetime was found to be in the range of 28.56 ns to 33.89 ns. Photocatalytic experiments were conducted for the degradation of methylene blue (MB) dye under sunlight irradiation in the presence of the In_2_O_3_ nanocubes. Among the five metal ion-doped samples, the Ni ion-doped In_2_O_3_ photocatalyst exhibited the highest degradation efficiency of 98% in 270 min of sunlight exposure. The high performance of Ni–In_2_O_3_ is due to its highest PL lifetime of 33.89 ns. The complete route for the degradation of MB dye was revealed by identifying the intermediates.

## Abstract 2

The objective of this work was to study the effect of transition metal ion doping (1 wt% of Mn, Fe, Co, Ni, and Cu) in indium oxide (In_2_O_3_) on its photocatalytic activity to degrade organic dyes, which are considered potential environment pollutants.

## Graphical abstract

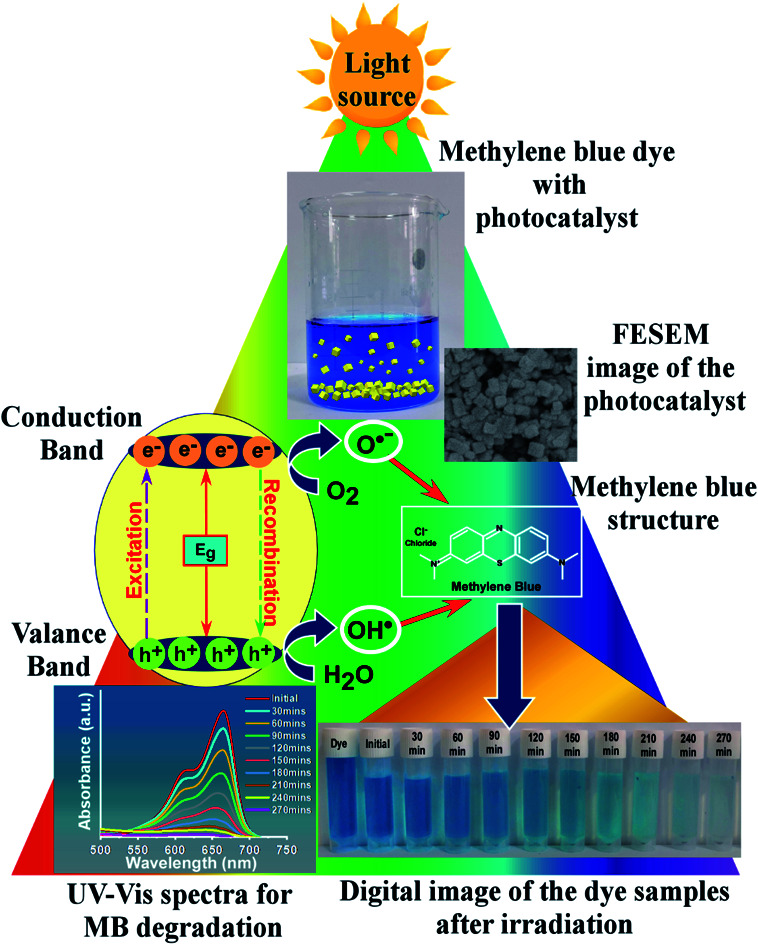

## 1. Introduction

Water is excessively used in all industries, which threatens the ecosystem when effluent water is let out in freshwater streams without proper treatment.^1^ In particular, the textile industry uses a huge quantity of water due to the nature of its work, and it also uses innumerable types of synthetic dyes, which are toxic to the environment,^2^ and thus has become a serious environmental issue. Thus, to overcome this environmental issue from the dying industry, various technologies/methods have been developed and utilized for the degradation of dyes. Among them, a widely used method is photocatalysis, also known as the advanced oxidation process. Nanotechnology has provided an advanced technique for the degradation of the dyes present in effluents using various nanomaterials as photocatalysts for photocatalysis. In a typical photocatalytic process, the photocatalyst is mixed with a dye effluent solution, which is then irradiated under a suitable light source such as UV, visible and sunlight. These catalyst materials react with the dye effluent under light irradiation and produce free radicals (*e.g.*, hydroxyl radicals), which are necessarily involved in the process of dye degradation to break the bonds of the organic substances (dyes) and convert them into either less harmful or safe products.^3^ Photocatalyst materials must possess some peculiar properties such as efficient light-absorbing capability, non-toxicity, high photostability, large carrier mobility and low charge recombination rate to exhibit a good photodegradation performance. An efficient process is not achieved when the catalyst materials have a large bandgap, which causes them to degrade dyes only in the presence of UV light, which is harmful and only makes up a small portion of the solar spectrum (about 4%). Thus, research interest has been shifted towards visible light (45% of the solar spectrum) active photocatalyst materials.^4^ For example, titania (TiO_2_), a well-known photocatalyst, has been used for many years, but it is active mainly in the UV region of the solar spectrum, which is a major drawback of this material.^5^ Light absorption capability is the other important characteristic of photocatalysts.^6^ The need for efficient photocatalysts is continuously increasing as the demand for a clean environment increases. Therefore, it is necessary to find highly efficient visible light active photocatalyst materials. Recently, semiconductor metal-oxides such as TiO_2_, ZnO, V_2_O_5_, CeO_2_, and SnO_2_ have been used as homogeneous photocatalysts, but they suffer enormously from a fast electron–hole recombination rate.^7–11^ In addition to simple oxides, composites such as In_2_O_3_–ZnO nanocomposites, layered double oxides and layered double hydroxides have also been successfully used for the photodegradation of methylene blue.^12,13^ Furthermore, photocatalysts have been tuned by various processes such as doping, surface decoration, and forming composites with homogeneous photocatalysts to yield heterogeneous photocatalysts with extended electron–hole recombination time.^14–17^ Recently, a new methodology has also been used in addition to the conventional photocatalysis. Instead of only photo-assisted reactions in photocatalysis, electrical assistance has also been applied such as electrocatalysis^12^ to degrade methylene blue. Another state-of-the-art technique is to understand the photocatalytic phenomena is measuring the photocurrent generated during photocatalysis.^18,19^ Additionally, the state-of-the-art materials such as single crystalline quaternary sulfide nanobelts,^20^ carbon dots^21^ and transition metal hydroxide nanosheet arrays on graphene^22^ were also reported for this application.

In consistent with the current demands, this work is aimed to prepare indium oxide (In_2_O_3_) photocatalysts *via* a hydrothermal method in pure form and doped with 1 wt% transition metals. This state-of-the-art material was designed to exhibit an enhanced photodegradation performance. The hydrothermal method was chosen in this work because, this method is relatively simple, and a well-defined morphology can be obtained. Moreover, this process is carried out at a high pressure in a reactor vessel, and therefore it can work very well at a relatively low temperature of around 100 °C. As a semiconductor metal oxide, the In_2_O_3_ possesses a bandgap energy in the range of 3.00 eV to 4.30 eV, it is active under visible light and shows high stability, non-toxicity and high light absorption capability.^23,24^ Transition metal doping was employed to change the physiochemical (optical and chemical) properties of the In_2_O_3_ photocatalyst, and five transition metals such as Mn, Fe, Co, Ni and Cu (1 wt%) were chosen for doping in In_2_O_3_ in the present work.^25^ The structure and optical properties of these photocatalysts were completely characterized using different techniques. In addition, their photocatalytic application was studied using methylene blue as a model organic dye, which is a common dye used in the dyeing industry.

## 2. Experimental procedure

### 2.1 Precursor materials

All procedures used the precursor chemicals as received without further purification. Indium(iii) chloride (Himedia, India) and ammonia solution (Merck) were used in the synthesis of In_2_O_3_. For transition metal ion doping, manganese(ii) acetate (Aldrich), cobalt(ii) nitrate, ferric nitrate, nickel(ii) nitrate (Himedia) and copper(ii) acetate (Merck) were used as the precursors of the transition metal ions Mn, Co, Fe, Ni and Cu, respectively. Methylene blue (Himedia) was the dye used in the photocatalytic experiments. Double distilled water was used as needed.

### 2.2 Synthesis of pure and metal ion-doped In_2_O_3_

The pure and transition metal ion-doped In_2_O_3_ photocatalysts were synthesized *via* the hydrothermal method. Indium(iii) chloride was used as the precursor and ammonia hydroxide as a precipitating agent. Briefly, 0.025 M indium(iii) chloride was dissolved in 80 mL distilled water and constantly stirred for 30 min until a clear homogeneous solution was obtained. Then 1 mL ammonia solution was added dropwise to the precursor solution under constant stirring for 30 min, forming a precipitate. The pH of the solution was measured to be 6 and 10 before and after the addition of ammonia solution, respectively. Then, 1 wt% of the appropriate transition metal ion precursor (that is, manganese(ii) acetate for Mn, cobalt(ii) nitrate for Co, ferric nitrate for Fe, nickel(ii) nitrate for Ni and copper(ii) acetate for Cu) was added to the above reaction solution. This mixture was transferred to a hydrothermal reaction chamber (a Teflon-coated stainless steel container), sealed completely and kept at 180 °C for 24 h in an oven. After completion of the reaction, the chamber was cooled to room temperature, and the formed precipitate was centrifuged, washed with water and acetone, and then dried at 80 °C. Initially, In(OH)_3_ was obtained using the above conditions. To obtain the transition metal-ion doped In_2_O_3,_ it was subsequently calcinated at 400 °C for 3 h. Pristine In_2_O_3_ was also prepared as a control sample following the same procedure.

### 2.3. Characterization

Thermogravimetric analysis was performed (Q600 SDT, TA Instruments) in an air atmosphere at a heating rate of 10 °C min^−1^. X-ray diffraction patterns were acquired on a powder X-ray diffractometer (Ultima-IV, Rigaku) using Cu-Kα radiation. Electron micrographs and X-ray energy dispersive spectra were acquired on a field emission scanning electron microscope (Hitachi, S-3400N) operated at 30 kV. UV-vis absorption spectroscopy was performed on a Shimadzu UV-3600 spectrophotometer. Steady state photoluminescence (PL) and time-resolved PL spectroscopy were conducted on a Fluorolog-FL3-11-Horiba spectrophotometer. All characterizations were performed at room temperature. Brunauer–Emmett–Teller (BET) specific surface area measurements were carried out on a Micrometrics Gemini VII 2390 *via* N_2_ absorption at 77 K.

### 2.4. Photocatalysis experiment

The photocatalytic performance of the transition metal ion-doped In_2_O_3_ photocatalysts was investigated through photodegradation studies on aqueous methylene blue dye (MB) solution under sunlight irradiation at ambient conditions. Firstly, 100 mL MB dye solution (aqueous) of 0.02 mM concentration was prepared and 50 mg photocatalyst (pure and transition metal ion-doped In_2_O_3_) was added to it and mixed homogeneously in the dark for 30 min to allow the dye molecules to be equilibrated with the photocatalyst. Then the reactant solution was exposed directly to sunlight continuously for 270 min, and at fixed intervals, the sunlight-exposed solution was collected and tested *via* UV-vis absorption analysis. The intermediate products formed during the pathway of photodegradation of MB dye under sunlight irradiation were studied *via* electrospray ionization mass spectroscopy (ESI-MS) (1260 Infinity series HPLC (Agilent) connected to LCMS-2020 (Shimadzu)). The analysis was carried out with the mobile phase of 37% acetonitrile and 0.1% formic acid, injection volume of 90 μL, Zorbax Eclipse Plus C18 (250 × 4.6 mm, 5 μm) column, electrospray ionization positive mode, and the *m*/*z* scan was performed in the range of 50–1000. To understand the dominant role of a particular active radical in photocatalysis, scavenger molecules were used, and this experiment was conducted using a similar method as that in our previous studies.^6,26^

## 3. Results and discussion

### 3.1 Differential scanning calorimetry (DSC)

**Fig. 1:**
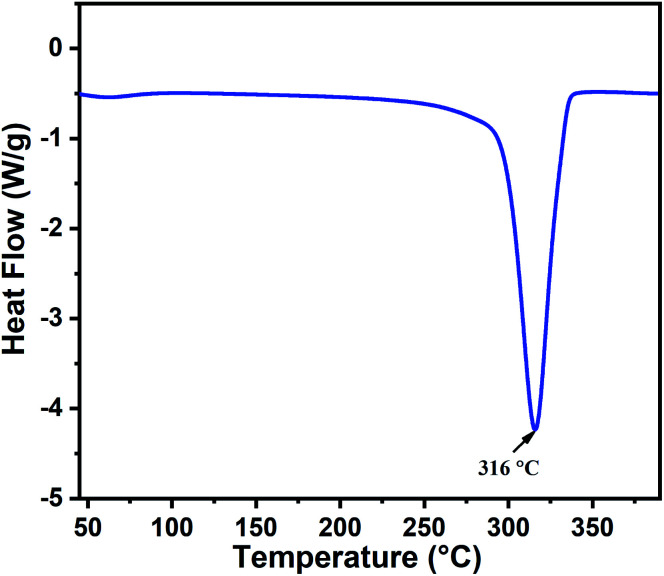
DSC thermogram of the as-prepared indium hydroxide, In(OH)_3_.

The thermal behavior of the as-prepared indium hydroxide (In(OH)_3_) sample was studied *via* DSC. Fig. 1 presents the DSC thermogram of In(OH)_3_, which shows a sharp endothermic peak at 316 °C, corresponding to the phase transformation of the as-prepared sample from the hydroxide phase to a well crystalline In_2_O_3_ phase. This temperature (316 °C) is attributed to the crystallization temperature of indium hydroxide to In_2_O_3_, which matches well with the reported values in the literature.^27^ Thus, based on the thermal analysis and the values reported in the literature, the calcination temperature for the as-prepared sample was fixed at 400 °C to obtain the crystalline phase of In_2_O_3_.

### 3.2 XRD analysis

**Fig. 2:**
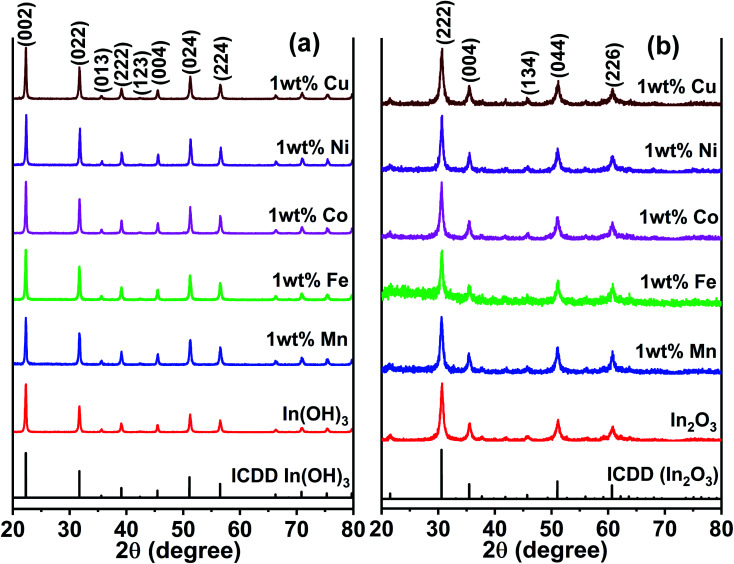
XRD patterns of pure and metal ion (Mn, Fe, Co, Ni and Cu) doped (a) as-prepared In(OH)_3_ and (b) In_2_O_3_. The latter was obtained by a calcination of the former at 400 °C.

Fig. 2a presents the XRD patterns of the as-prepared In(OH)_3_ with different doped metal ions and that the of the 400 °C calcinated In_2_O_3_ are shown in Fig. 2b. The as-prepared samples of In(OH)_3_ (Fig. 2a) show X-ray reflections at 2*θ* values of 22.28°, 31.71°, 39.10°, 45.46°, 51.19°, and 56.49°, which match well with the cubic phase of In(OH)_3_ possessing a space group symmetry of *Im*3̄ (ICDD #98-001-7283). In contrast, the reflections observed at 30.58°, 35.46°, 45.68°, 51.02°, and 60.66° in Fig. 2b correspond to a cubic phase of In_2_O_3_ whose structural symmetry is governed by the *Ia*3̄ space group (ICDD #98-000-6517). Thus, the complete structural transformation from hydroxide phase into cubic In_2_O_3_ is obvious when the former was calcinated at 400 °C. Further, it is also clear that no impurity phases are present in any of the metal ion-doped In_2_O_3_. Hereafter, all the studies were performed only on the metal ion-doped In_2_O_3_.

The lattice parameters were calculated using the standard formula for the cubic phase and the obtained lattice parameters are listed in Table 1. It can be observed that the variation in the lattice parameter was small because the doping concentration of the transition metal ions was just 1 wt%. When the metal ion concentration was increased to 3 wt%, a secondary phase was formed, and therefore, this study was limited to 1 wt% of transition metal ion doping. The XRD results for In_2_O_3_ with different concentrations of dopant ions are presented in Fig. S1 of the ESI,† where the impurity phases are marked with an asterisk. These impurity/secondary phases were not identified. The average crystallite size of these samples was estimated using the Scherrer equation,^28^ and the crystallite sizes were observed to vary in the range of 12 nm to 18.7 nm (listed in Table 1). In particular, the size varied in the range of 12.0 to 14.1 nm for the Cu-, Ni-, Co- and Mn-doped In_2_O_3_, whereas Fe doping alone yielded a size of 18.7 nm. The ionic radius of In^3+^ is 80 pm, whereas that of Cu^2+^, Ni^2+^, Co^3+^, Fe^3+^ and Mn^2+^ was 77, 70, 60, 60, and 70 pm, respectively. Since all the dopant ions possess a lower ionic radius, besides introducing a slight strain in the In_2_O_3_ lattice upon doping, they did not influence the crystallite size significantly. The strain values due to doping were estimated from the Williamson–Hall plot of the XRD data and are presented in Table T1 of the ESI.† The strain values for In_2_O_3_ varied in the range of 4.42 × 10^−3^ to 10.71 × 10^−3^ when doped with these metal ions. Therefore, the influence of the crystallite size on the physicochemical properties of the metal ion-doped In_2_O_3_ was ruled out in this work.

**Table 1:** Estimated average crystallite sizes, lattice parameters and the band gap energies of the pure and 1 wt% transition metal ion-doped In_2_O_3_

Sample	Average crystallite size, *d* (nm)	Lattice parameter, *a* (Å)	Band gap energy (eV)
In_2_O_3_	12.0	1.4862	3.00
1 wt% Cu–In_2_O_3_	12.5	1.4857	2.97
1 wt% Ni–In_2_O_3_	13.4	1.4855	2.99
1 wt% Co–In_2_O_3_	14.1	1.4844	2.97
1 wt% Fe–In_2_O_3_	18.7	1.4851	2.94
1 wt% Mn–In_2_O_3_	14.1	1.4846	2.97

### 3.3 Raman spectroscopy studies

**Fig. 3:**
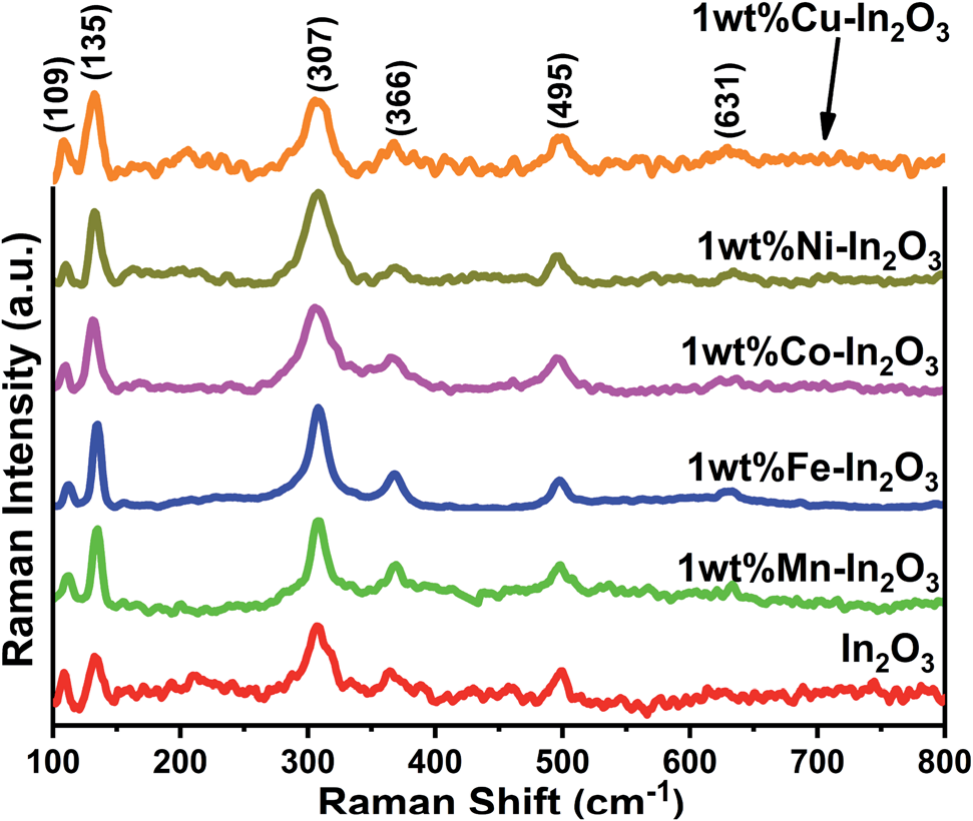
Raman spectra of pure and 1 wt% transition metal ion Mn, Fe, Co, Ni, and Cu-doped In_2_O_3_.

The Raman spectra for the pure and transition metal ion-doped In_2_O_3_ are presented in Fig. 3. The vibrational modes of the cubic In_2_O_3_ are given as *Γ* = 4A_g_ + 4E_g_ + 14F_2g_.^29^

All the Raman modes shown in Fig. 3 agree well with that of the cubic-structured In_2_O_3_, and the observed Raman modes at 109, 135, 307, 366, 495 and 631 cm^−1^ confirm that the structural symmetry of In_2_O_3_ is governed by the *Ia*3̄ space group.^30^ In particular, the vibrations with A_g_ and E_g_ symmetry are Raman active but infrared inactive and the Raman modes are observed at 135 cm^−1^ and 631 cm^−1^, respectively. These A_g_ and E_g_ modes originated from the In–O vibrations. The other two Raman bands observed at 307 cm^−1^ and 366 cm^−1^ are assigned to In–O–In vibrations, which also signify the existence of oxygen vacancies in the In_2_O_3_ structure.^31^ The width (full width at half maximum, FWHM) of the band at 307 cm^−1^ was estimated by fitting that particular peak using a Lorentz function, and found to vary between 16.1 and 30.96 (see Fig. S2 of the ESI†). The FWHM of the band 307 cm^−1^ generally correlates with the crystallite size of In_2_O_3_. The FWHM was observed to be high, that is, the peak is broad for the lower grain sizes and increased thereafter. However, the width of the Fe-doped In_2_O_3_ was found to be lowest among the samples, corroborating its highest crystallite size of 18.7 nm.

### 3.4 Microstructure analysis by FESEM

**Fig. 4:**
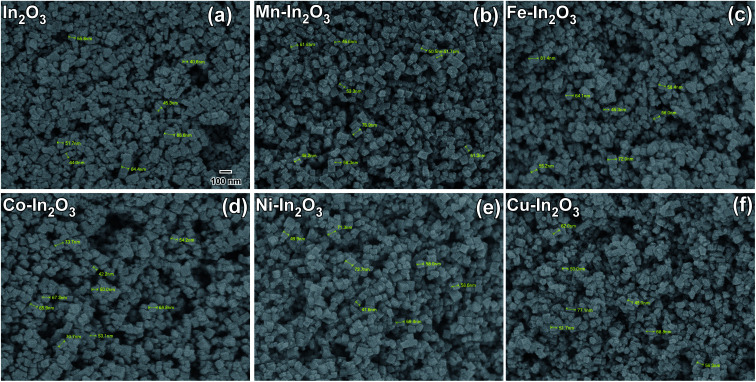
Field emission SEM micrographs (secondary electrons were used) of (a) pure In_2_O_3_, (b) 1 wt% Mn–In_2_O_3_, (c) 1 wt% Fe–In_2_O_3_, (d) 1 wt% Co–In_2_O_3_, (e) 1 wt% Ni–In_2_O_3_ and (f) 1 wt% Cu–In_2_O_3_. The particle size distribution of each of the In_2_O_3_ nanocube samples is presented in Fig. S3 of the ESI.† The markings on the micrographs were used to obtain the particle size estimation. Scale bar given in (a) is the same for all the figures.

The morphologies of the pure and Mn, Fe, Co, Ni, and Cu metal ion-doped In_2_O_3_ were studied *via* FE-SEM and the SEM micrographs acquired using secondary electrons are presented in Fig. 4. By careful analysis of the FE-SEM micrographs, the morphology of the pure and metal ion-doped In_2_O_3_ was observed as cube-shaped, and therefore they are referred to as nanocubes. The shape of these nanocubes is generally uniform and monodispersed. The particle size distribution was obtained by measuring the particle size of several particles from the SEM images. About 75 to 100 particles were counted in each case to obtain their respective size distribution histograms, which are presented in Fig. S3 of the ESI.† The size distribution histograms were fitted with a Gaussian function to obtain the average size, which was observed to vary in the range of 41.7 nm to 52.1 nm. These nanocubes are well discernible from one another, which means that they are not agglomerated. Additionally, no observable changes are occurred in the morphology of the In_2_O_3_ nanocubes upon the addition of metal dopants. Further, the elemental composition was studied through XEDS, as shown in Fig. S4 (ESI†), which show the presence of In and O elements. However, in the metal-doped In_2_O_3_, the presence of In and O and the respective metal ions was observed. Since the concentration of metal ion doping was only 1 wt%, the peaks in the XEDS spectra corresponding to the metal atoms are not predominant, but observed to be significant.

### 3.5 Diffused reflectance spectroscopy analysis

**Fig. 5:**
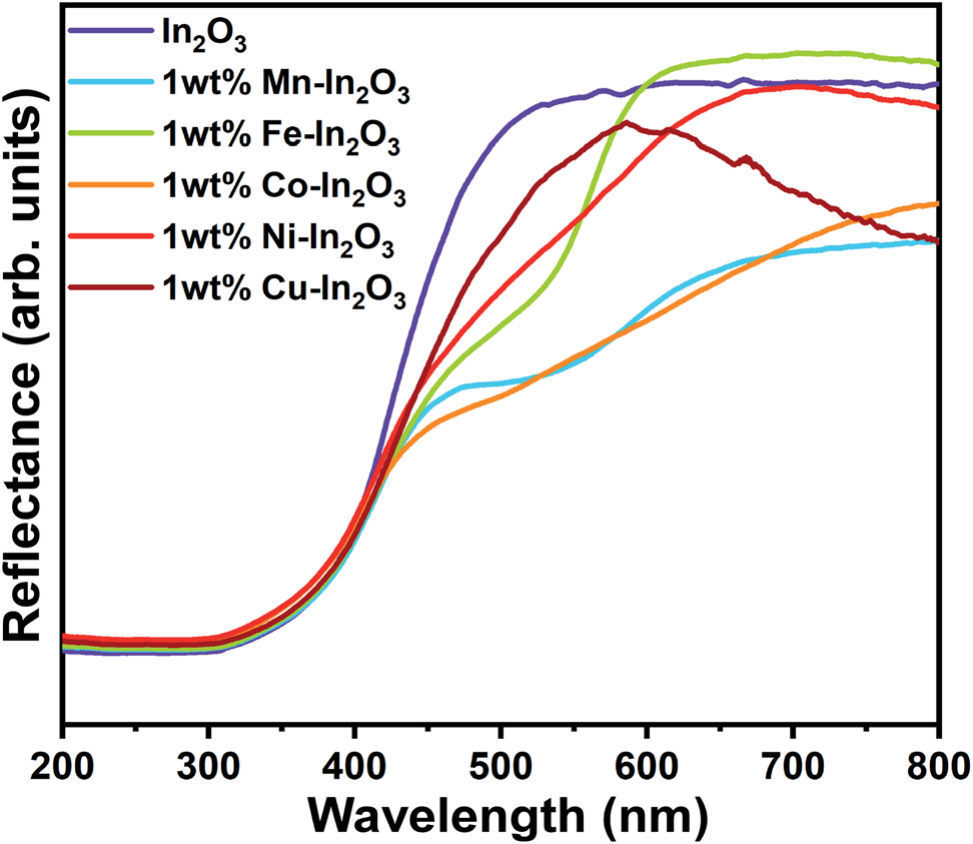
UV-vis diffused reflectance spectra for the pure and transition metal ion-doped In_2_O_3_.

Because the In_2_O_3_ photocatalyst samples possess a nanocube microstructure, their surface would be rough enough to allow specular reflection to occur when any optical beam hit them, and therefore diffused reflectance spectroscopy (DRS) was used to study the optical absorption properties of these samples. The DRS spectra for the pure and 1 wt% Mn, Fe, Co, Ni, and Cu ion-doped In_2_O_3_ nanocubes acquired in the wavelength range of 200 to 800 nm are presented in Fig. 5.

The optical band gap energy of the metal ion-doped In_2_O_3_ was estimated by plotting the DRS spectra (see Fig. 5) using the Kubelka–Munk function,^32^ which is expressed as follows:1
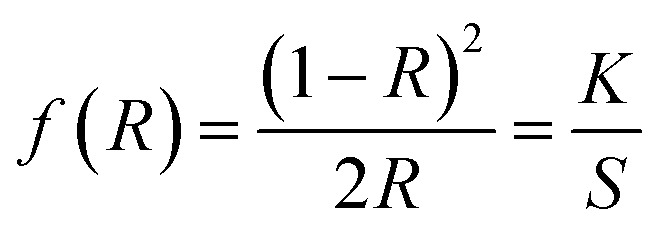
where *R* is the reflectance, and *K* and *S* are the absorption and reflection coefficients, respectively. The Kubelka–Munk function plots for all the In_2_O_3_ samples are presented in Fig. S3 of the ESI.† The flat regions of the reflectance profiles were extrapolated to the *x*-axis to provide the band gap energy, which was determined to vary from 2.94 eV to 3.00 eV. Even though the crystallite size variation was small for each sample, a slight dependence on the band gap energy on crystallite size was observed. It is very common in nanomaterials with a lower grain size to have a higher band gap energy due to the physical attribute known as “quantum confinement”. In this case, pure In_2_O_3_ with a crystallite size of 12.0 nm possesses a band gap of 3.00 eV, whereas the Fe-doped In_2_O_3_ with a crystallite size of 18.7 nm has a band gap of 2.94 eV. The other samples show a clear correlation with their respective crystallite size. However, the variation in the bandgap values for each sample is significantly small.

### 3.6 Photoluminescence emission studies

**Fig. 6:**
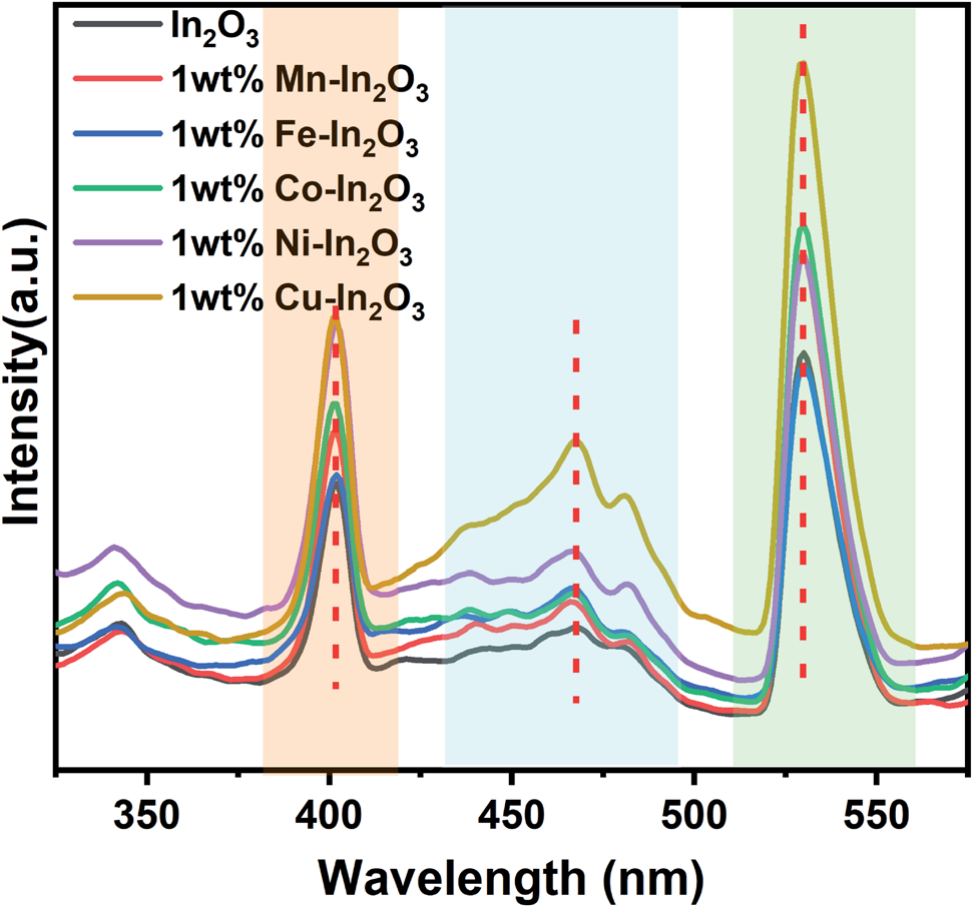
Photoluminescence emission spectra of pure and transition metal ion-doped In_2_O_3_. Three distinct regions of emission are marked with different colour shades.

Fig. 6 presents the photoluminescence (PL) spectra of the pure and transition metal ion-doped In_2_O_3_ acquired at 300 nm excitation. Three well-defined emission peaks can be observed in three wavelength regions, as shaded in different colors (in Fig. 6) for all the In_2_O_3_ samples, including the metal-ion doped samples. It should be noted that bulk In_2_O_3_ does not show emission, as reported by Ohhata *et al.*^33^ However, many researchers have reported the PL emission in In_2_O_3_, mostly due to the presence of oxygen defects. For In_2_O_3_ thin films, only one emission at 637 nm was observed, and this orange emission was attributed to the presence of oxygen deficiencies or defects.^34^ Another group reported a broad PL emission with a centre at 470 nm when measured at room temperature.^35^ Nanocuboids of In_2_O_3_ have already been reported in the literature.^36^ Two emissions were commonly reported for In_2_O_3_ at 405 nm and 570 nm when they were prepared with a nanofibre morphology.^37^ However, in the single crystalline nanowires, two PL peaks with a maximum at 380 nm were also reported.^38^ We observed PL emission at three different wavelengths (405, 470 and 530 nm). Based on the reports available in the literature, there are two possibilities for obtaining an emission for In_2_O_3_. One is due to the quantum confinement effect when the particle size of In_2_O_3_ is smaller or about the same as its critical Bohr radius. The second possibility is the presence of oxygen defects. Liang *et al.*^39^ clearly ruled out the possibility of the PL emission from quantum confinement because the diameter of the nanofibers they prepared was far away from the critical Bohr radius for In_2_O_3_, which is 2.14 nm. Consistent with their discussion, the particle sizes of our In_2_O_3_ varied from 12.0 nm to 18.7 nm, which is much higher than the critical Bohr radius of In_2_O_3_, and therefore, the size-induced PL emission due to the quantum confinement effect is also ruled out in our case. Therefore, the observed PL emissions at 405, 470 and 530 nm can be attributed to oxygen vacancies. Upon doping transition metal ions in the In_2_O_3_ lattice, there are reports stating a gradual increase in the number of oxygen vacancies.^36,37^ A blue emission was observed from In_2_O_3_ at 405 and 470 nm, which are attributed to the radiative recombination of an electron and hole (*V*_In_ and *V*_O_) in In_2_O_3_, respectively. The third emission appeared at 530 nm, which falls in the yellow-orange region and its origin is the recombination of electrons in singly ionised oxygen vacancies with holes in the valence band or doubly ionised oxygen vacancies. In particular, the In_2_O_3_ cube nanostructures favour the generation and existence of a large quantities of oxygen vacancies.^40^ Another study reported three PL emissions at 430, 480 and 520 nm for In_2_O_3_ nanoparticles.^41^ It is also interesting to note that the emission peak positions (marked in Fig. 6 with vertical dotted red lines) of these three emissions are not shifted upon doping of the transition metal ions. Therefore, it can also be concluded that the same mechanism of PL emission occurs in all the In_2_O_3_ samples, which is obviously due to oxygen defects.

### 3.7 PL lifetime analysis

**Fig. 7:**
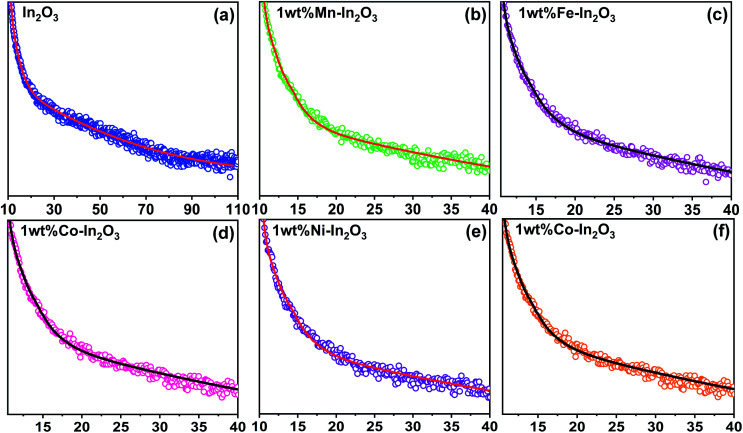
Time-resolved photoluminescence decay curves for the pure and transition metal ion-doped In_2_O_3_. Discrete points are the experimental data and continuous lines were fit to the data using a triexponential function given in ref. 43.

The time-resolved photoluminescence (TRPL) lifetime decay profiles for the pure and transition metal ion-doped In_2_O_3_ are presented in Fig. 7. All the TRPL decay profiles were fitted with the triexponential function^42^ and the average PL lifetime (*τ*_avg_) was obtained using a formula^43^ containing the weightage amplitudes *A*_1_, *A*_2_ and *A*_3_ and decay time constants *τ*_1_, *τ*_2_ and *τ*_3_.

The lifetime components and their relative amplitudes obtained through triexponential fitting of the TRPL decay profiles are listed in Table 2. The lifetime component *τ*_1_ lies in the range of 1.95–2.99 ns, *τ*_2_ varies from 27.5 ns to 35 ns, and *τ*_3_ varies from 0.03 ns to 0.59 ns. However, the average lifetime (*τ*_avg_) for all the samples was in the range of 26.50–33.89 ns. The average lifetime for 1 wt% Ni–In_2_O_3_ was 33.89 ns, which is the highest among pure In_2_O_3_ and the other samples. Consequently, Ni ion doping significantly increased the charge separation and the enhanced lifetime of the charge carriers in the photocatalyst material, making it very suitable for application in photodegradation.

**Table 2:** Time constants and their relative amplitudes for the pure and transition metal ion-doped In_2_O_3_ obtained by fitting the time-resolved photoluminescence decay profiles using the triexponential function

Sample	*τ*_1_ (ns)	*τ*_2_ (ns)	*τ*_3_ (ns)	*A*_1_ (%)	*A*_2_ (%)	*A*_3_ (%)	Average lifetime *τ*_avg_ (ns)
In_2_O_3_	2.21	29.75	0.03	4.32	9.08	86.6	28.56
1 wt% Mn–In_2_O_3_	1.95	28.10	0.03	6.29	12.8	80.91	27.06
1 wt% Fe–In_2_O_3_	2.38	30.60	0.32	21.26	53.43	25.31	29.61
1 wt% Co–In_2_O_3_	2.11	27.50	0.14	18.79	40.58	40.63	26.50
1 wt% Ni–In_2_O_3_	2.99	35.00	0.59	22.15	61.25	16.6	33.89
1 wt% Cu–In_2_O_3_	2.22	31.53	0.22	22.09	50.08	27.83	30.53

### 3.8 Photocatalytic studies

#### 3.8.1 Effect of catalyst loading

The optimum quantity of catalyst loading required to achieve the best photocatalytic degradation is a very crucial factor to consider. Therefore, firstly, a set of experiments was carried out to determine the optimal quantity of catalyst loading, which would also reduce the wastage or extra usage of catalyst material in the process of photocatalysis, and consequently, the total process would become less expensive. The experiments were performed by varying the amount of photocatalyst (that is pure In_2_O_3_) loading including 25 mg, 50 mg, 75 mg and 100 mg and the concentration of MB dye was kept constant for all the trials. For the photocatalytic experiments, pure In_2_O_3_ was dispersed in MB dye solution and stirred in the dark for 30 min, and then the homogenized and equilibrated solution was then irradiated under sunlight for 150 min. The sunlight-exposed solution was collected at 30 min intervals and spectroscopically analyzed.

**Fig. 8:**
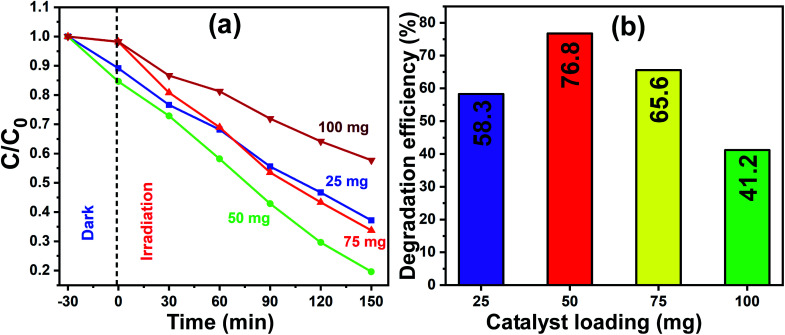
(a) Normalized concentration profiles for MB dye degradation with different catalyst (pure In_2_O_3_) loadings of 25 mg, 50 mg, 75 mg and 100 mg under sunlight irradiation. (b) Corresponding photocatalytic efficiency for each catalyst loading for the exposure time of 150 min.

Fig. 8a presents the linearity of the photodegradation reaction for the various catalyst loadings of 25 mg, 50 mg, 75 mg and 100 mg and the corresponding degradation efficiencies are shown as a bar graph in Fig. 8b. The degradation efficiency is increased from 58% to 76% as the In_2_O_3_ loading increased from 25 mg to 50 mg, and a further increase in the catalyst loading to 100 mg led to a decrease to 41%. The decrease in the degradation efficiency at a higher catalyst loading is due to two reasons, one is the greater light scattering and the second is the screening effects. Since the number of photocatalyst particles available is more, they lead to the heavy scattering of light and the other reason is the screening effect of this heavy scattering. In addition, an obvious change in the nature of the reactant dye solution was observed, which looked “clear” when the catalyst loading was less, whereas it became turbid for a higher catalyst loading. It is understandable that when the system is turbid, this would not allow sunlight to pass through, which reduced the effective degradation of the dye solution.^42^ In this work, 50 mg catalyst loading showed a maximum degradation of MB dye, and therefore 50 mg of In_2_O_3_ was considered to be the optimum quantity and used subsequently herein.

#### 3.8.2 Photocatalytic studies on MB

**Fig. 9:**
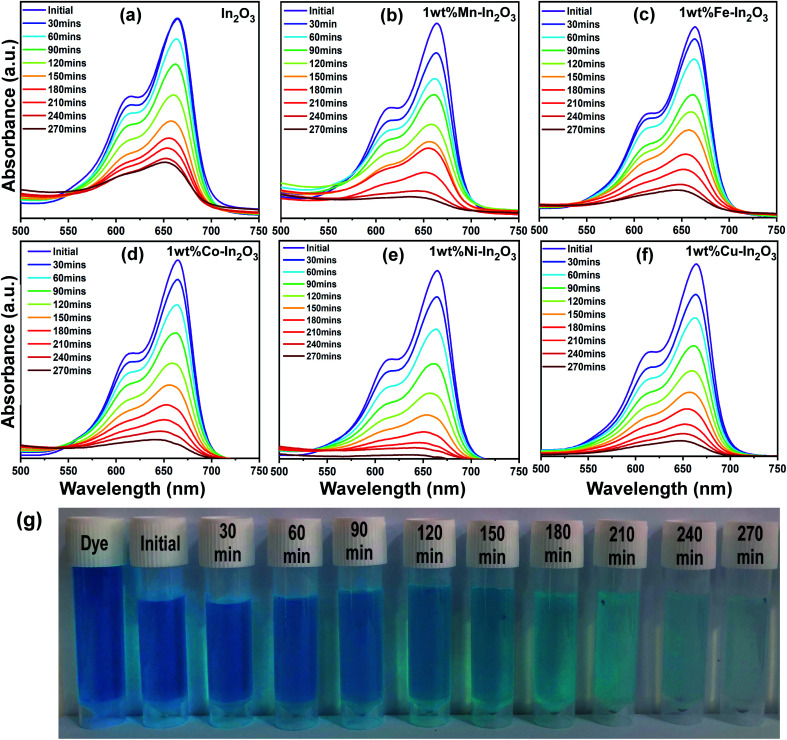
(a–f) UV-vis absorption spectra for pure and Mn, Fe, Co, Ni, and Cu-doped In_2_O_3_ photocatalysts showing the evolution of absorption intensity of MB dye upon direct sunlight irradiation for the total irradiation time of 270 min, respectively. The decrease in intensity shows a decrease in the content of MB upon sunlight irradiation. (g) Digital images showing the color of the dye solution after periodic photocatalytic degradation of MB in the presence of 1 wt% Ni–In_2_O_3_ under sunlight for 270 min.

After the photocatalyst loading was optimized, photocatalytic experiments were carried out with the optimal catalyst loading of 50 mg in 100 mL aqueous dye solution under direct sunlight irradiation for 270 min. The absorption spectra of the irradiated solutions for different time intervals are shown in Fig. 9, which clearly show a decrease in absorption intensity with an increase in sunlight irradiation time.

**Fig. 10:**
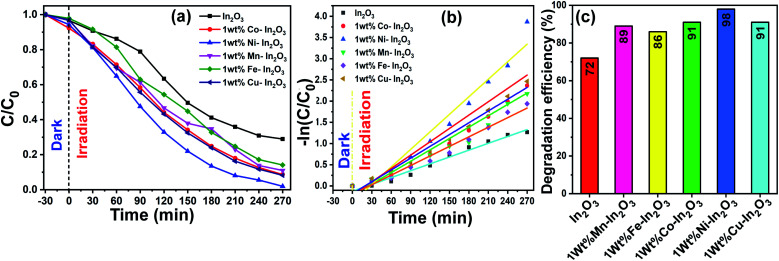
(a) *C*/*C*_0_*versus* of irradiation time, (b) −ln(*C*/*C*_0_) *versus* reaction time, where the experimental data is shown by discrete points and the linear fit to the data is shown by continuous lines, and (c) efficiency bar graph for pure and transition metal ion-doped (Mn, Fe, Co, Ni, and Cu) In_2_O_3_ photocatalysts for the degradation of MB dye. The continuous lines in (a) are a guide to the eyes.

The degradation efficiency of the photocatalysts was obtained from the UV-vis spectral evolution using the formula,2
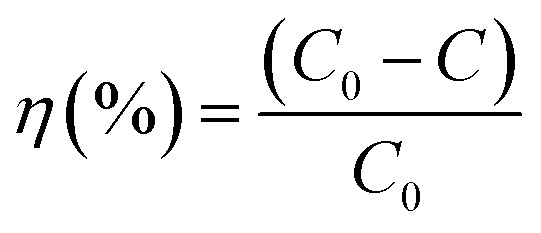
where *C*_0_ and *C* are the initial and final MB concentration, respectively. The degradation efficiencies of the pure In_2_O_3_, Mn, Fe, Co, Ni and Cu-doped In_2_O_3_ were determined to be 71%, 89%, 86%, 91%, 98% and 91%, respectively. A bar diagram showing the maximum degradation by all these photocatalysts is presented in Fig. 10b. Then, using the *C*_0_ and *C* values obtained from the UV-vis absorption spectra, −ln(*C*/*C*_0_) *versus* sunlight irradiation time was plotted, as shown in Fig. 10c. A good linearity of the photocatalytic reaction was obtained with respect to irradiation time, confirming that the photocatalyst undergoes a pseudo-first-order kinetic reaction for the degradation of dye molecules. Additionally, the reaction rate constants for the photodegradation reactions were calculated from the slope of the linear fitting to the data in Fig. 10c, and the rate constant *k* was determined to be 0.00538 min^−1^, 0.00798 min^−1^, 0.00753 min^−1^, 0.00893 min^−1^, 0.01378 min^−1^, 0.00926 min^−1^ using the pure and Mn, Fe, Co, Ni and Cu-doped In_2_O_3_ photocatalysts respectively.

In the literature, In_2_O_3_ has been widely studied as a visible light active photocatalyst.^44^ Using solid-state reaction, a series of metal ion-doped MIn_2_O_4_ (M = Ca, Sr, and Ba) photocatalysts were reported to exhibit higher activity for MB degradation in 120 min under visible light, and their performance was reported to be much higher than that of the commercial Degussa P25 powder.^45^ CaIn_2_O_4_ was synthesized *via* a solution-combustion method, which took only 90 min to decompose MB under visible light irradiation.^46^ Another research group reported that a core-shell-like coupled composite In_2_O_3_–CaIn_2_O_4_, which was synthesized *via* sequential calcination, showed better visible light-induced photodegradation of MB than the single component CaIn_2_O_4_. This enhanced and quick activity within 40 min was attributed to the selective charge separation and efficient charge transport at the interface of In_2_O_3_ and CaIn_2_O_4_.^47^ In another study, a core–shell like nanocomposite of In_2_O_3_@Ba_2_In_2_O_5_, which was prepared *via* the chemical impregnation method, degraded MB completely in just 30 min under visible light.^48^ The sol–gel method-synthesized composite In_2_O_3_–TiO_2_ was studied for the degradation of Alizarin Red using different contents of In_2_O_3_. However, the composite with a 3% In_2_O_3_ content showed the highest photocatalytic activity with 99% degradation of Alizarin Red in 75 min.^49^ A new material system, TiO_2_ co-doped with Ag and In_2_O_3_, containing a three–component junction was produced. These Ag–In_2_O_3_–TiO_2_ nanocomposites exhibited the highest activity for the degradation of Rhodamine B (Rh-B) completely in 45 min compared to pure TiO_2_, single-doped TiO_2_ and P25 Degussa powder.^50^ The In_2_O_3_ tuned to have a bandgap in the visible region (3.55–3.75 eV) and fabricated by the co-precipitation method, when doped with Eu^3+^, showed higher efficiency than pure In_2_O_3_, but still the efficiency of the Eu^3+^-doped In_2_O_3_ was only 45% in 120 min under sunlight irradiation.^51^ Another group has developed three types of nanostructured In_2_O_3_ with different morphologies such as nanocubes, nanoplates, and porous microspheres, which were used for the degradation of perfluorooctanoic acid (PFOA), and the degradation rates of the nanocubes, nanoplates and porous microspheres were 17.3, 41.9, and 74.7 times faster than that by P25 TiO_2_.^52^ Rhombohedral-structured In_2_O_3_ nanocrystals synthesized *via* the solvothermal method degraded 92% of Rh-B (rhodamine-B) and MB in reaction times of 4 h and 3 h, respectively.^53^ Thus, compared to the other photocatalysts reported in the literature, the Ni-doped In_2_O_3_ in this study shows a comparatively good degradation in a reasonable time. To visualize the degradation performance of the Ni-doped In_2_O_3_, the degraded MB dye solutions after different periods of sunlight irradiation were digitally imaged, as presented in Fig. 9g, where the decoloration of the dye with respect to irradiation time can be observed.

Another crucial factor influencing the photodegradation performance is the surface area of the samples. Thus, to understand this parameter, BET surface area measurements were done selectively on two samples, namely pure In_2_O_3_ and 1 wt% Ni–In_2_O_3_. The BET surface area was determined to be 48.14 (±0.2881) m^2^ g^−1^ and 50.81(±0.1249) m^2^ g^−1^ for the pure In_2_O_3_ and 1 wt% Ni–In_2_O_3_, respectively. The surface area was slightly higher in the case of Ni-doped In_2_O_3_, which could also have assisted in enhancing its photodegradation performance compared to that of pure In_2_O_3_.

#### 3.8.3 Understanding the active radicals in photocatalysis

**Fig. 11:**
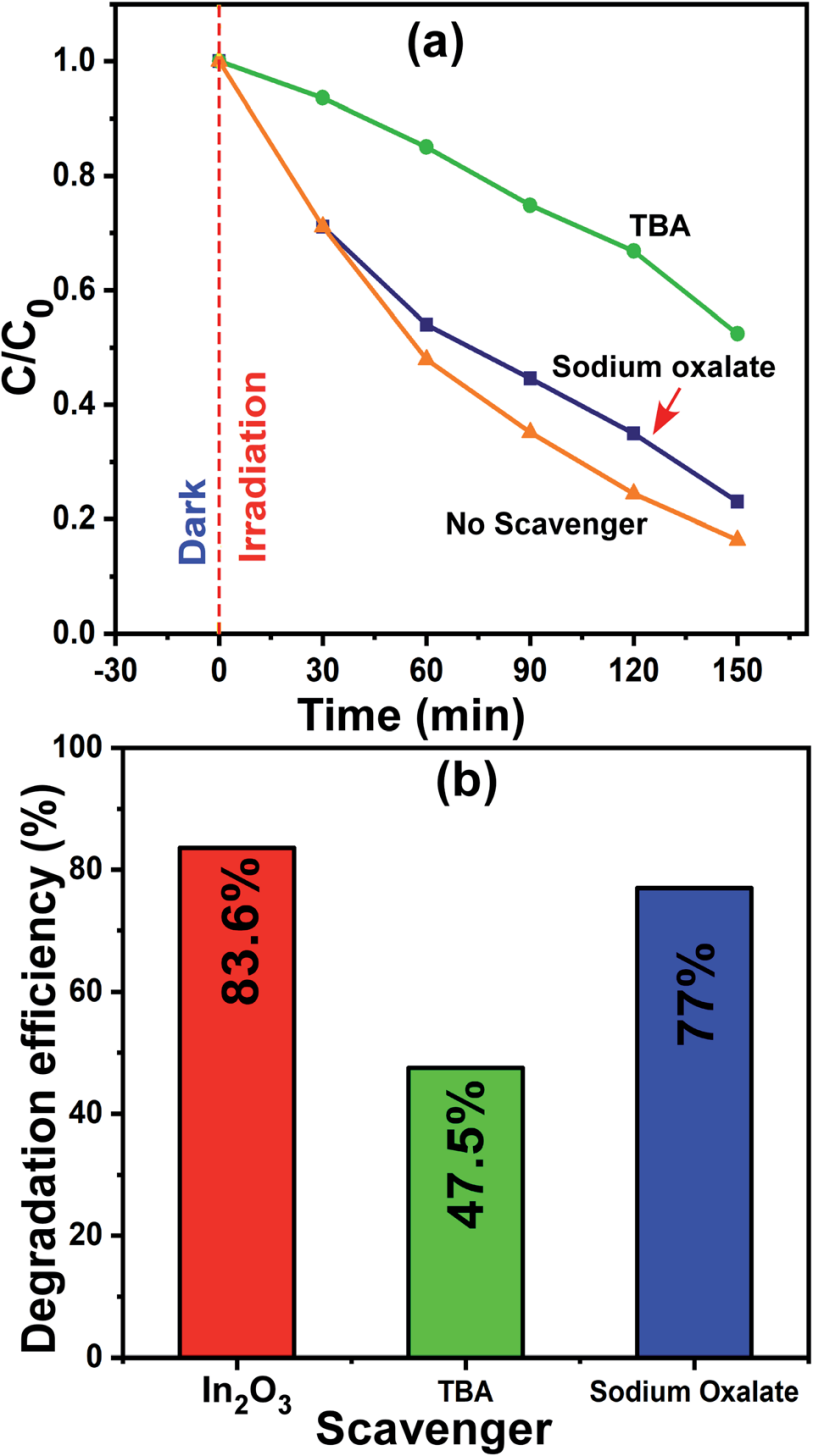
(a) Change in relative concentration for different times of sunlight irradiation and (b) maximum photodegradation efficiencies for MB dye in the presence of scavengers SO and TBA molecules.

The most common reactive species that participate in photocatalysis are electrons, holes (h^+^) and hydroxyl radicals (˙OH). On many occasions, one or more of them work for the degradation of the dye molecules. Thus, is very important to understand the type of species participating in the degradation, although this is challenging. However, the use of appropriate scavenger molecules in the regular photocatalytic experiments can identify the specific reactive species. Thus, we used the scavenger molecules of sodium oxalate (SO) for holes (h^+^) and *tert*-butyl alcohol (TBA) for hydroxyl radicals (˙OH). Additionally, all the experimental conditions were maintained as that in the regular photocatalytic experiments. In this case, the 1 wt% Ni–In_2_O_3_ photocatalyst with the best efficiency was used, and a detailed procedure of this experiment was reported in our previous publication.^6,26^ The reaction mixture containing MB solution, 50 mg of 1 wt% Ni–In_2_O_3_ and scavenger molecules SO and TBA was irradiated with sunlight for 150 min and the irradiated solutions were analyzed *via* UV-vis absorption spectroscopy. The absorption spectra are shown in Fig. S4 of the ESI.† The normalized concentration *C*/*C*_0_*versus* time plot and the corresponding maximum photodegradation efficiency are presented in Fig. 11a and b, respectively. With no scavengers added, the degradation efficiency was 83.6%, whereas the efficiencies were 77% and 47.5% when TBA and SO were used, respectively. Among these three cases, the degradation of MB was observed to be the lowest when TBA was added, which implies that the maximum contribution to the photodegradation is provided by hydroxyl radicals (˙OH). Since TBA scavenges ˙OH radicals, the overall photodegradation efficiency was reduced significantly. Therefore, conclusively, the hydroxyl radicals (˙OH) radicals played a dominant role in the photodegradation of the MB system.^54^

#### 3.8.4 Identification of intermediate degradation products

**Fig. 12:**
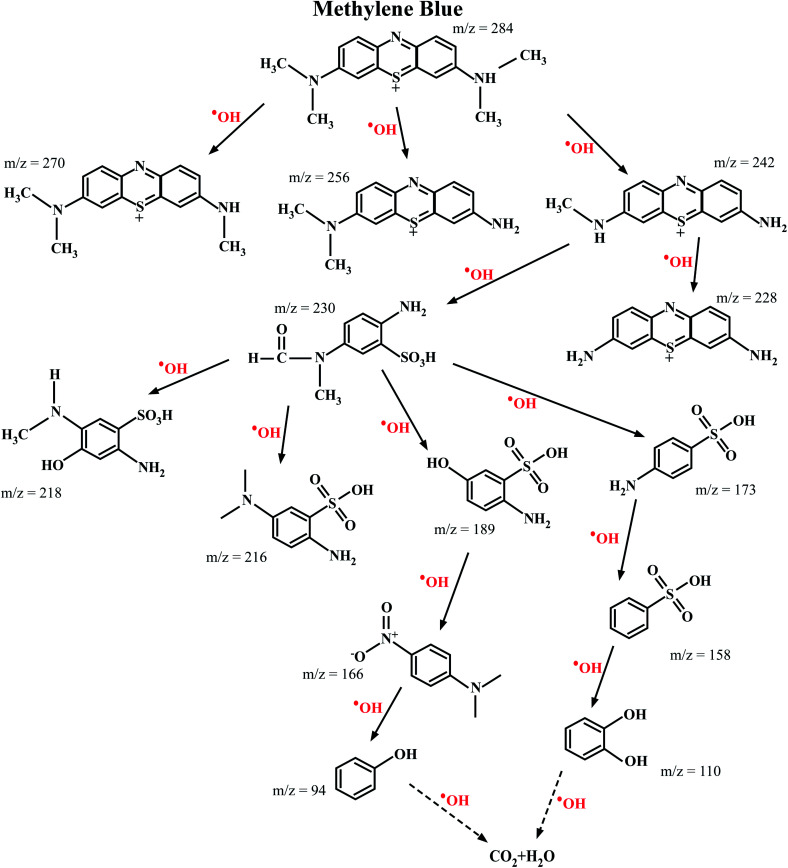
Most probable intermediate compounds identified using the specific mass to charge ratio formed during the photodegradation of MB dye under sunlight radiation traced through ESI-MS spectroscopy. The photocatalyst used for this experiment was the 1 wt% Ni–In_2_O_3_.

The ESI-MS spectroscopy analysis gives the exact values of the mass to charge ratio (*m*/*z*) of the most probable molecules present in a given solution, and therefore it was used to analyze the degradation pathway of MB dyes in this work. This particular experiment was carried out for the photocatalytic degradation of MB with Ni-doped In_2_O_3_ as the photocatalyst. The total degradation obtained for the period of 0 min to 270 min of sunlight irradiation was considered. With the obtained results, the degradation pathway (that is, the by-products/intermediates) for MB dye under sunlight irradiation was elucidated, as shown in Fig. 12. The ESI-MS spectra acquired for 0, 60, 120, 240 and 270 min of irradiation are presented in Fig. S5 of the ESI.†

The photocatalytic reaction proceeded with the disintegration of the MB dye molecules upon irradiation with sunlight in the presence of 1 wt% Ni–In_2_O_3_ photocatalyst. The MB dye has an *m*/*z* value of 284, and the peak in the initial spectrum (Fig. S5 in the ESI†) at 0 min shows a peak at 278, which corresponds to MB. The signature peak of MB was present in the 60 min and 180 min irradiated solutions; however, with a longer irradiation time, it either disappeared or became less intense, which clearly shows the degradation of the dye. In the case of 180 min and 240 min, many low-intensity peaks are appeared, which imply the presence of many disintegrated molecules in the solution. However, at the last irradiation time (270 min), only one high intense peak with a low *m*/*z* value was present and much less low-intensity peaks are appeared, indicating that there were less molecules too in a lower quantity. This clearly shows the complete degradation of MB into fragments of very small *m*/*z*, which are not expected to be harmful. In summary, the initially used MB dye solution was demineralised by 98% in 270 min in the presence of the photocatalyst into several *m*/*z* values such as 270 (Azure-A), 256 (Azure-B), 242 (Azure-C), 230 (2-amino-5-(*N*-methyl formamide) benzene sulfonic acid), 228 (thionin), 218 (2-amino-5-(methylamino)hydro benzene sulfonic acid), 216 (2-amino-5-dimethyl amino benzene sulfonic acid), 189 (2-amino-5-hydroxy-benzene sulfonic acid), 173 (4-amino-benzene sulfonic acid), 166 (dimethyl-4-nitro-phenyl-amine), 158 (benzene sulfonic acid), 110 (catechol or *p*-dihydroxy benzene), and 94 (phenol) at various stages of sunlight irradiation. As studied from the radical scavenging test, these degradation reactions occurred in the presence of active hydroxyl radicals (OH), which were generated by the reduction reaction occurring in the valence band of the semiconductor photocatalyst, that is Ni-doped In_2_O_3_. These hydroxyl radicals interact with the MB molecules and degraded them it into less harmful or safe molecules with lower *m*/*z* values.^55–60^

### 3.9 Mechanism of photodegradation

**Fig. 13:**
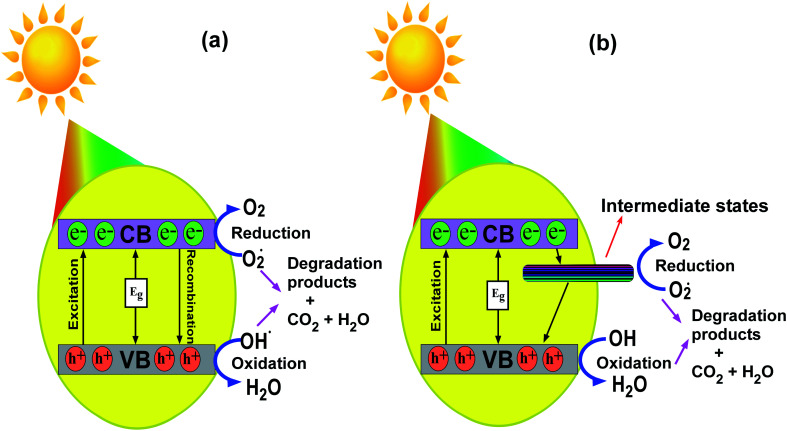
Schematic representation portraying the photodegradation mechanism in MB using (a) pure and (b) transition metal ion-doped In_2_O_3_ nanocubes.

The mechanism for the photodegradation of MB by the transition metal ion-doped In_2_O_3_ photocatalyst is illustrated based on the band diagram depicted in Fig. 13. Upon sunlight irradiation, the reactions are initiated by forming an electron–hole pair on the surface of the photocatalyst.

The energy supplied by sunlight assists electrons to jump to the conduction band from the valence band, leaving a hole in the latter. These photogenerated electrons and holes on the surface of the In_2_O_3_ photocatalyst react with electron acceptors such as O_2_ molecules, which are adsorbed on the surface of In_2_O_3_, and produce the superoxide radical O_2_˙^−^ through a reduction process. Simultaneously, the holes in the valence band combine with the OH^−^ radicals and oxidize them to form hydroxyl radicals, ˙OH. The formed hydroxyl radicals ˙OH and superoxide radical anion (O_2_˙^−^) species are the major oxidisers capable of degrading the molecular structure of MB/organic pollutants into harmless products, as shown in Fig. 13. In this particular case, the existence of oxygen vacancies and the incorporation of transition metal ions in the In_2_O_3_ lattice act as a reservoir for electrons or holes and booster for the interfacial recombination, leading to superior photocatalytic degradation.^26^ It should be noted that pure In_2_O_3_ does have a direct transition of the excited state electrons to any intermediate states available between the conduction and valence bands, and therefore this may lead to faster recombination, and thus it showed a lower photodegradation efficiency.

### 3.10 Recyclability and stability of the photocatalysts

**Fig. 14:**
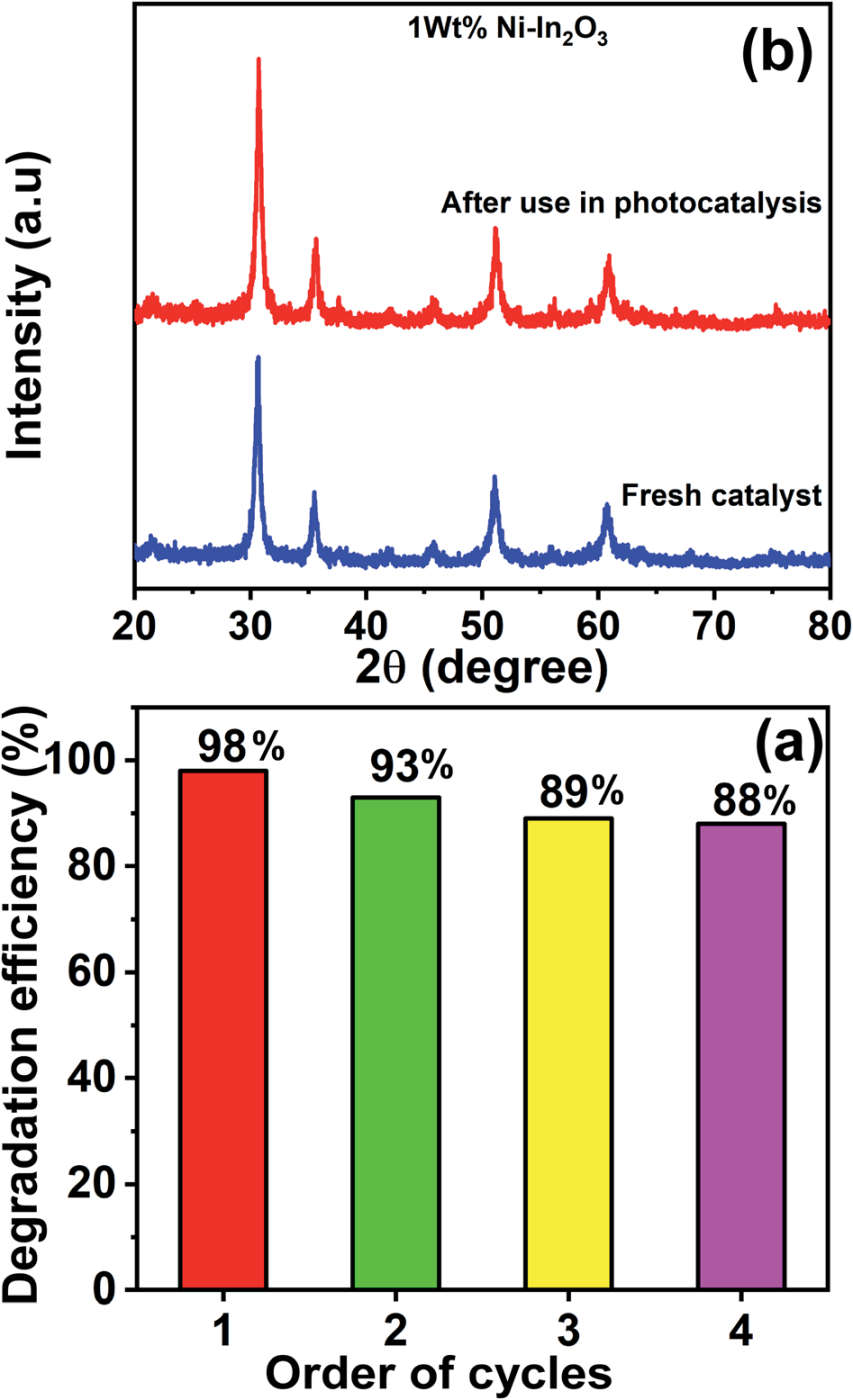
(a) Efficiency bar graph for the photodegradation of MB by 1 wt% Ni–In_2_O_3_ under sunlight irradiation for four continuous photocatalytic cycle experiments. (b) Comparison of XRD patterns of the fresh photocatalyst 1 wt% Ni–In_2_O_3_ with that acquired after use for four cycles of photocatalytic experiment.

The best performing photocatalyst sample (that is, the 1 wt% Ni–In_2_O_3_) was chosen for the recyclability and stability tests. These tests were carried out for four continuous cycles under sunlight irradiation using the same experimental conditions. After each cycle, the catalyst material was extracted by centrifugation and used in the subsequent cycle. The acquired UV-vis absorption spectra for these four cycles are presented in Fig. S6 of the ESI,† and the degradation efficiencies of the “recovered and re-used” catalyst materials are listed in Fig. 14a. The first cycle showed an efficiency of 98%, whereas that for the second, third and fourth cycles is 93%, 89% and 88%, respectively. Evidently, the first cycle showed the highest efficiency and there was a slight reduction in the subsequent cycles. This reduction in efficiency is not attributed to photobleaching (which is a common phenomenon in materials such as ZnO) of the photocatalyst, but because of the slight loss of photocatalyst during recovery from the previous cycles.

Fig. 14b shows the XRD patterns of the 1 wt% Ni–In_2_O_3_ photocatalyst acquired before and after its use in four cycles of photocatalytic experiment. Both patterns look similar with no obvious change, indicating that the crystallinity of the photocatalyst was well preserved even after four times use in the photocatalytic experiments and it was highly stable under sunlight irradiation and the other experimental conditions.

To understand the enhanced performance of the photocatalysts in the present study, they were compared with other photocatalysts reported in the literature for the degradation of methylene blue dye. The comparison is presented in Table 3. Although the photocatalysts and the excitation sources are different, the photocatalysts reported in this study showed much better performance for the degradation of MB dye (98% degradation).

**Table 3:** Photodegradation of methylene blue by different types of photocatalysts compared with the reported values

Photocatalyst	Photodegradation efficiency (%)	Light source	Ref.	Year reported
In_2_O_3_	88	UV	44	2010
V_2_O_5_/BiVO_4_	92	Visible	61	2011
Fe–Cd–ZnO	82	Visible	62	2011
ZnO	78	UV	15	2012
ZnO-nanorods	85	UV	63	2013
V_2_O_5_	89	Visible	9	2014
Au–V_2_O_5_@ZnO	83	UV-visible	64	2014
Fe_2_O_3_	22	Sunlight	65	2015
ZnO–SnO_2_^−^	30	UV	16	2016
Transition metal ion doped In_2_O_3_	98	Sunlight	This work	—

It should be noted that the present study has good commercial significance because many industries, particularly the textile industry, use organic dyes for colouring fabrics. Since methylene blue is one of the major dyes used in these industries, the present transition metal-doped In_2_O_3_ photocatalysts are highly useful for this purpose. The mechanism of degradation is clearly understood together with complete structural analysis in this work, which will increase its scope. Moreover, these photocatalysts are highly stable for repeated use, and therefore highly economical for commercial use.

## 4. Conclusions

In summary, pure and five different 1 wt% transition metal ion-doped, *i.e.* Mn, Fe, Co, Ni, and Cu, In_2_O_3_ photocatalysts were synthesized *via* the hydrothermal method. All of them possessed a nanocube morphology. They were formed with cubic structure, and no other impurity phases were detected. Since the doping content was 1 wt%, no significant change in the structure was observed for all the metal ion-doped samples. Similarly, the bandgap of the pure In_2_O_3_ was observed to increase slightly with an increase in average particle size; however, the variation was in the range of 2.99 to 2.94 eV. The PL lifetime was observed to be higher in the doped In_2_O_3_, where Ni doping showed the highest lifetime among them. The photocatalytic performances of the photocatalysts were tested for the degradation of aqueous MB dye solution under direct sunlight irradiation for 270 min. The transition metal ion doping resulted in an improved performance compared to that of pure In_2_O_3_. Among them, the 1 wt% Ni–In_2_O_3_ photocatalyst showed the maximum efficiency of 98%. This enhanced photodegradation by Ni–In_2_O_3_ was attributed to its higher PL lifetime, which facilitated a lower recombination rate of electron–hole pairs. Additionally, the metal ion doping introduced additional intermediate energy levels within the forbidden gap, which helped to increase the lifetime of the photogenerated charge carriers, and thus increased the photocatalytic activity. The reaction rate constant was observed to be maximum for Ni doping, and hence it had emerged as the best photocatalyst. These materials were proven to be very stable for many cycles of photocatalytic applications, with consistent efficiency and re-usability. The route for the photodegradation of MB dye was traced by understanding the intermediate-formed fragmented molecules. The mechanism of improved photocatalytic activity was explained well based on the band diagram. From a practical point of view, these materials are relatively inexpensive and highly efficient for the degradation of dye molecules, and thus can be used as successful photocatalysts for a sustainable environment.

## Conflicts of interest

This work does not have any conflict of interest to declare.

## Supplementary Material

NA-003-D0NA00694G-s001

## References

[cit1] Hassaan M. A., Nemr A. (2017). Am. J. Environ Sci Eng..

[cit2] Khataee A. R., Kasiri M. B. (2010). J. Mol. Catal. A: Chem..

[cit3] Chan S. H. S., Wu T. Y., Juan J. C., Teh C. Y. (2011). J. Chem. Technol. Biotechnol..

[cit4] Wang Y., Liu L., Xu L., Cao X., Li X., Huang Y. (2014). Nanoscale.

[cit5] Hashimoto K., Irie H., Fujishima A. (2006). Jpn. J. Appl. Phys..

[cit6] Santhosh Kumar J., Sadishkumar V., Arun T., Thangadurai P. (2018). Mater. Sci. Semicond. Process..

[cit7] Wang Y., Zhu Y., Zhao X., Yang X., Li X., Chen Z., Yang L., Zhu L. (2015). Surf. Coat. Technol..

[cit8] Wang C., Gao Y., Wang L., Li P. (2017). Phys. Status Solidi A.

[cit9] Roy A., Pradhan M., Ray C., Sahoo R., Dutta S., Pal T. (2014). CrystEngComm.

[cit10] Saravanan R., Joicy S., Gupta V. K., Narayanan V., Stephen A. (2013). Mater. Sci. Eng., C.

[cit11] Bhattacharjee A., Ahmaruzzaman M., Devi T. B., Nath J. (2016). J. Photochem. Photobiol., A.

[cit12] Zhao J., Ge S., Pan D., Pan Y., Murugadoss V., Li R., Xie W., Lu Y., Wu T., Wujcik E. K., Shao Q., Mai X., Guo Z. (2019). J. Electrochem. Soc..

[cit13] Liu X., Shao Q., Zhang Y., Wang X., Lin J., Gan Y., Dong M., Guo Z. (2020). Colloids Surf., A.

[cit14] Li B., Xu Y., Rong G., Jing M., Xie Y. (2006). Nanotechnology.

[cit15] Kajbafvala A., Ghorbani H., Paravar A., Samberg J. P., Kajbafvala E., Sadrnezhaad S. K. (2012). Superlattices Microstruct..

[cit16] DaSilva F., Lopes O. F., Catto A. C., Jr A. (2016). RSC Adv..

[cit17] Gnanasekaran L., Hemamalini R., Ravichandran K. (2015). J. Saudi Chem. Soc..

[cit18] Yang C., Dong W., Cui G., Zhao Y., Shi X., Xia X., Tang B., Wang W. (2017). RSC Adv..

[cit19] Jayaraj S. K., Thangadurai P. (2020). J. Mol. Liq..

[cit20] Wu L., Wang Q., Zhuang T.-T., Li Y., Zhang G., Liu G.-Q., Fan F.-J., Shi L., Yu S.-H. (2020). Nat. Commun..

[cit21] Fang J., Debnath T., Bhattacharyya S., Döblinger M., Feldmann J., Stolarczyk J. K. (2020). Nat. Commun..

[cit22] Lu K.-Q., Li Y.-H., Zhang F., Qi M.-Y., Chen X., Tang Z.-R., Yamada Y. M. A., Anpo M., Conte M., Xu Y.-J. (2020). Nat. Commun..

[cit23] Guilmeau E., Pelloquin D. (2008). J. Magn. Magn. Mater..

[cit24] Chandradass J., Bae D. S., Kim K. H. (2011). Adv. Powder Technol..

[cit25] Xu X. X., Cui Z. P., Gao X., Liu X. X. (2014). Dalton Trans..

[cit26] Santhosh Kumar J., Thangadurai P. (2018). J. Environ. Chem. Eng..

[cit27] Guo S., Zhang X., Hao Z., Gao G., Li G., Liu L. (2014). RSC Adv..

[cit28] Monshi A., Foroughi M. R., Monshi M. R. (2012). World J. Nano Sci. Eng..

[cit29] Kranert C., Schmidt-grund R., Grundmann M. (2014). Phys. Status Solidi RRL.

[cit30] Berengue O. M., Rodrigues A. D., Dalmaschio C. J., Lanfredi A. J. C., Leite E. R., Chiquito A. J. (2010). J. Phys. D: Appl. Phys..

[cit31] Gan J., Lu X., Wu J., Xie S., Zhai T., Yu M., Zhang Z., Mao Y., Wang S. C. I., Shen Y. (2013). Sci. Rep..

[cit32] Myrick M. L., Simcock M. N., Baranowski M., Morgan S. L., Mccutcheon J. N. (2011). Appl. Spectrosc. Rev..

[cit33] Ohhata Y., Shinoki F., Yoshida S. (1979). Thin Solid Films.

[cit34] Lee M. S., Choi W. C., Kim E. K., Kim C. K., Min S. D. K. (1996). Thin Solid Films.

[cit35] Liang C., Meng G., Lei Y., Fritz P., Zhang L. (2001). Catalytic Growth of Semiconducting In_2_O_3_ Nanofibers. Adv. Mater..

[cit36] Liu G. (2011). Int. J. Electrochem. Sci..

[cit37] Papageorgiou P., Zervos M., Othonos A. (2011). Nanoscale Res. Lett..

[cit38] Min B., Lee J.-S., Keem K., Kim H., Jeong D.-Y., Cho K., Kim S. (2006). Synthesis of Single Crystalline In_2_O_3_ Nanowires and Their Photoluminescence Characteristics. Jpn. J. Appl. Phys..

[cit39] Liang C., Meng G., Lei Y., Fritz P., Zhang L. (2001). Adv. Mater..

[cit40] Peng X. S., Meng G. W., Zhang J., Wang X. F., Wang Y. W., Wang C. Z., Zhang L. D. (2002). J. Mater. Chem..

[cit41] Zhou H., Cai W., Zhang L. (1999). Appl. Phys. Lett..

[cit42] Tayade R. J., Natarajan T. S., Bajaj H. C. (2009). Ind. Eng. Chem. Res..

[cit43] Gaurav A., Beura R., Kumar J. S., Thangadurai P. (2019). Mater. Chem. Phys..

[cit44] Talebian N., Nilforoushan M. R. (2010). Thin Solid Films.

[cit45] Tang J., Zou Z., Katagiri M., Kako T., Ye J. (2004). Catal. Today.

[cit46] Ding J., Sun S., Bao J., Luo Z., Gao C. (2009). Catal. Lett..

[cit47] Chang W. K., Koteswara Rao K., Kuo H. C., Cai J. F., Wong M. S. (2007). Appl. Catal., A.

[cit48] Chang W. K., Wu Y. S., Tzeng C. Y., Lin A. Y. (2009). J. Alloys Compd..

[cit49] Rodriguez-Gonzaleza V., Paraguay-Delgadob F., Garcia-Montelongoa X., Torres-Martineza L. M., Gomezc R. (2008). J. Ceram. Process. Res..

[cit50] Yang X., Wang Y., Xu L., Yu X., Guo Y. (2008). J. Phys. Chem. C.

[cit51] Anand K., Thangaraj R., Kumar P., Kaur J., Singh R. C. (2015). AIP Conf. Proc..

[cit52] Li Z., Zhang P., Shao T., Wang J., Jin L., Li X. (2013). J. Hazard. Mater..

[cit53] Yin J., Cao H. (2012). Inorg. Chem..

[cit54] Rajeshwari S., Kumar J. S., Rajendrakumar R. T., Ponpandian N., Thangadurai P. (2018). Mater. Res. Express.

[cit55] Chaudhari M., Gawal M., Sane K., Sontakke M., Nemade R. (2018). Res. Chem. Intermed..

[cit56] Lee J., Tri N., Wook S., Jung E., Hong S. (2015). Mater. Chem. Phys..

[cit57] Yang C., Dong W., Cui G., Zhao Y., Shi X., Xia X., Tang B., Wang W. (2017). RSC Adv..

[cit58] Houas A., Lachheb H., Ksibi M., Elaloui E. (2001). Appl. Catal., B.

[cit59] Wang X., Han S., Zhang Q., Zhang N., Zhao D. (2018). MATEC Web Conf..

[cit60] Jia P., Tan H., Liu K., Gao W. (2018). Mater. Res. Bull..

[cit61] Su J., Zou X., Li G., Wei X., Yan C., Wang Y., Zhao J., Zhou L., Chen J. (2011). J. Phys. Chem. C.

[cit62] Neena D., Kondamareddy K., Bin H., Lu D., Kumar P., Dwivedi R. K., Pelenovich V., Zhao X., Gao W., Fu D. (2018). Sci. Rep..

[cit63] Saravanan R., Gupta V. K., Narayanan V., Stephen A. (2013). J. Mol. Liq..

[cit64] Yin H., Yu K., Song C., Huang R., Zhu Z. (2014). ACS Appl. Mater. Interfaces.

[cit65] Liu X., Chen K., Shim J., Huang J. (2015). J. Saudi Chem. Soc..

