# Supplementary material for: Transition metal ion-doped In2O3 nanocubes: investigation of their photocatalytic degradation activity under sunlight
Source: Nanoscale Adv. 2020 Nov 19;3(2):471–85. doi: 10.1039/d0na00694g (PMC9418826; doi:10.1039/d0na00694g)
Supplement: NA-003-D0NA00694G-s001 [file NA-003-D0NA00694G-s001.pdf]

Electronic Supplementary Material (ESI) for Nanoscale Advances.
This journal is © The Royal Society of Chemistry 2020

Transition metal ion-doped In2O3 nanocubes: Investigations of their photocatalytic degradation activity under sunlight

Velayutham Shanmuganathan[1], Jayaraj Santhosh Kumar[1], Raman Pachaiappan[2], Paramasivam Thangadurai[1,*]

[1]*Centre for Nanoscience and Technology, Pondicherry University, Kalapet, Pondicherry, 605014, India*
[2]*Department of Biotechnology, School of Bioengineering, SRM University, Kattankulathur - 603 203, Tamilnadu, India*
**Email:* thangadurai.p@gmail.com/thangaduraip.nst@pondiuni.edu.in

Table T1: Strain obtained from the Williamson-Hall fitting of the XRD data.

S.No.	Sample	Strain, ε
1.	In_2O_3	4.75×10^{-3}
2.	Cu-In_2O_3	5.21×10^{-3}
3.	Ni-In_2O_3	10.71×10^{-3}
4.	Co-In_2O_3	4.85×10^{-3}
5.	Fe-In_2O_3	9.98×10^{-3}
6.	Mn-In_2O_3	4.42×10^{-3}

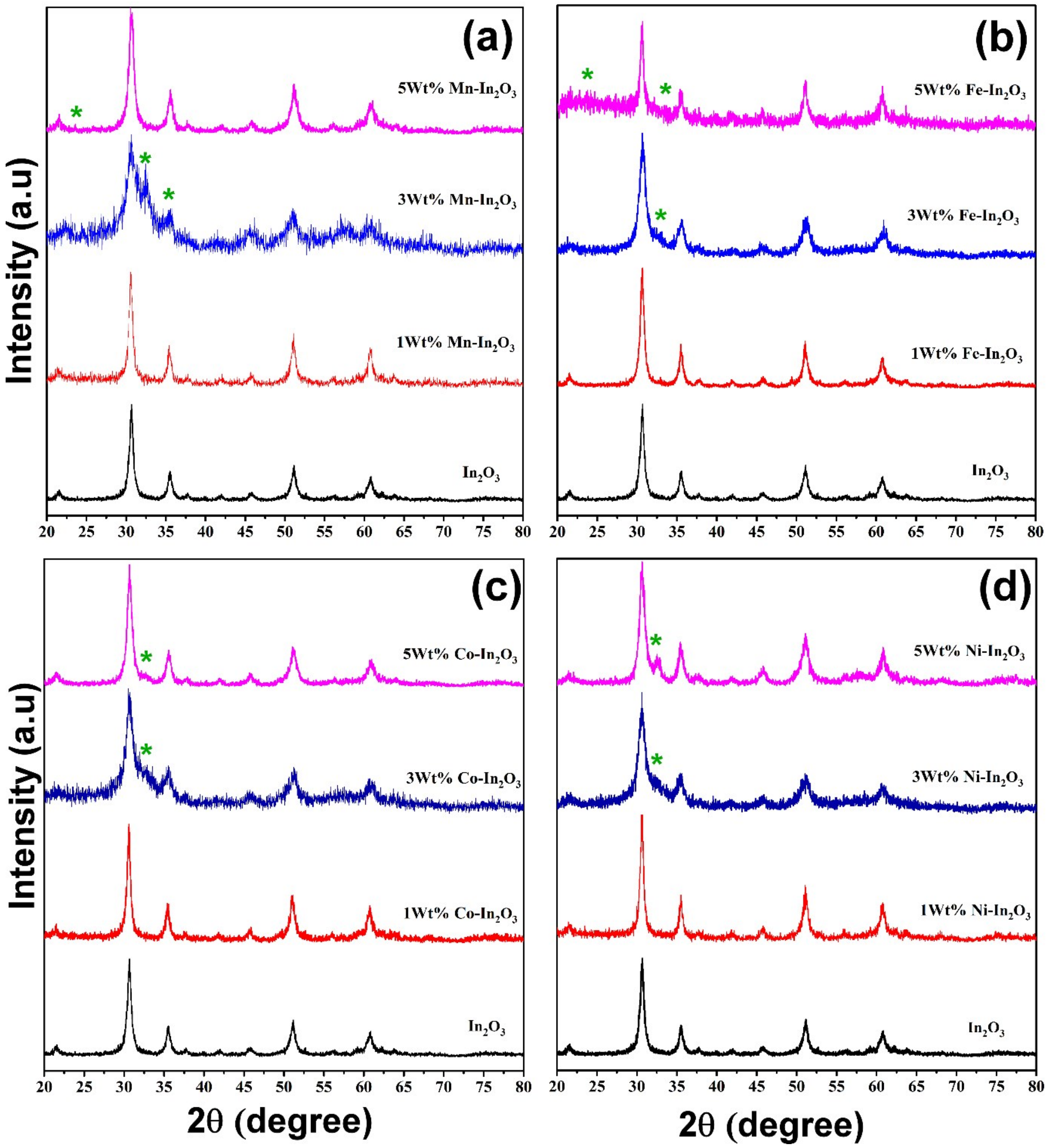

Figure S1. The XRD patters of the In_2O_3 for different concentration of the dopant ions. Asterisk marks the impurity phases in each sample when the concentration is increased. The XRD patterns for Mn, Fe, Co and Ni ion doped In_2O_3 are given respectively in (a), (b), (c) and (d).

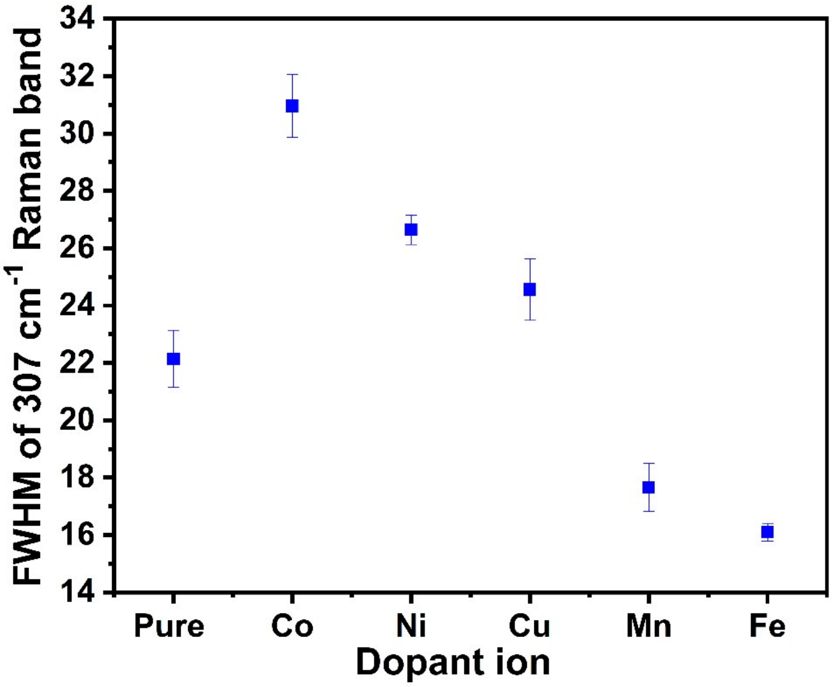

Figure S2. Change in FWHM of the Raman band at 307 cm^{-1} in In_2O_3 for various doping ions.

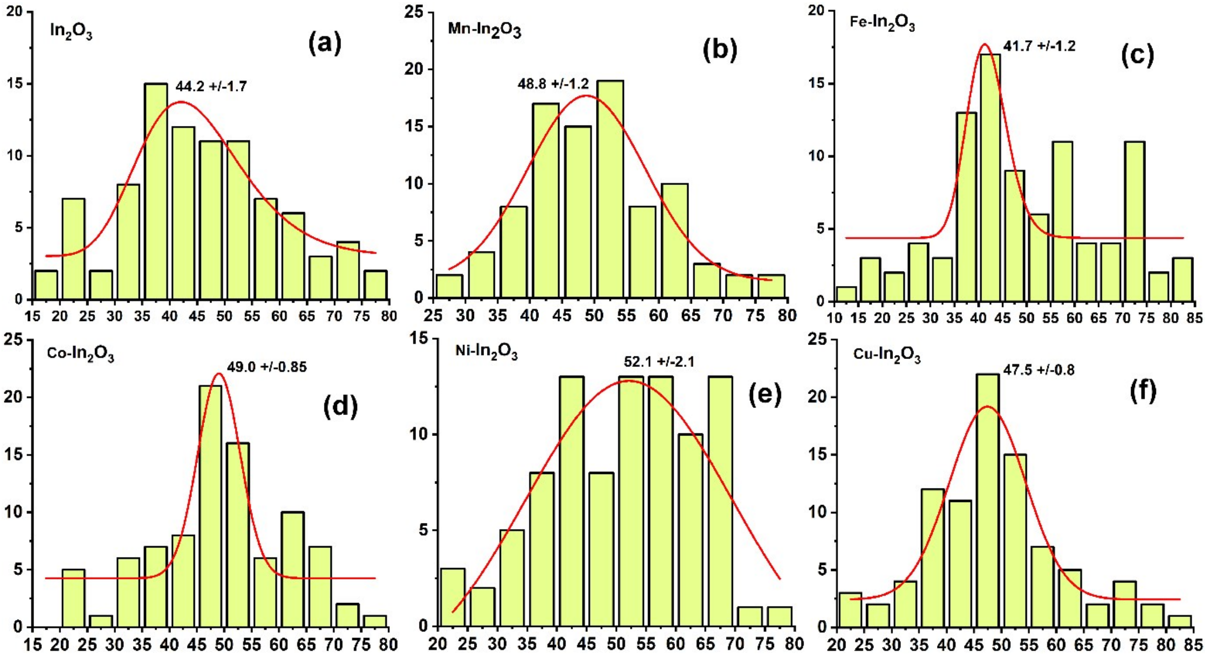

Figure S3. Particle size distribution in (a) In_2O_3, (b) 1wt% Mn-In_2O_3, (c) 1wt% Fe-In_2O_3, (d) 1wt% Co-In_2O_3, (e) 1wt% Ni-In_2O_3 and (f) 1wt% Cu-In_2O_3.

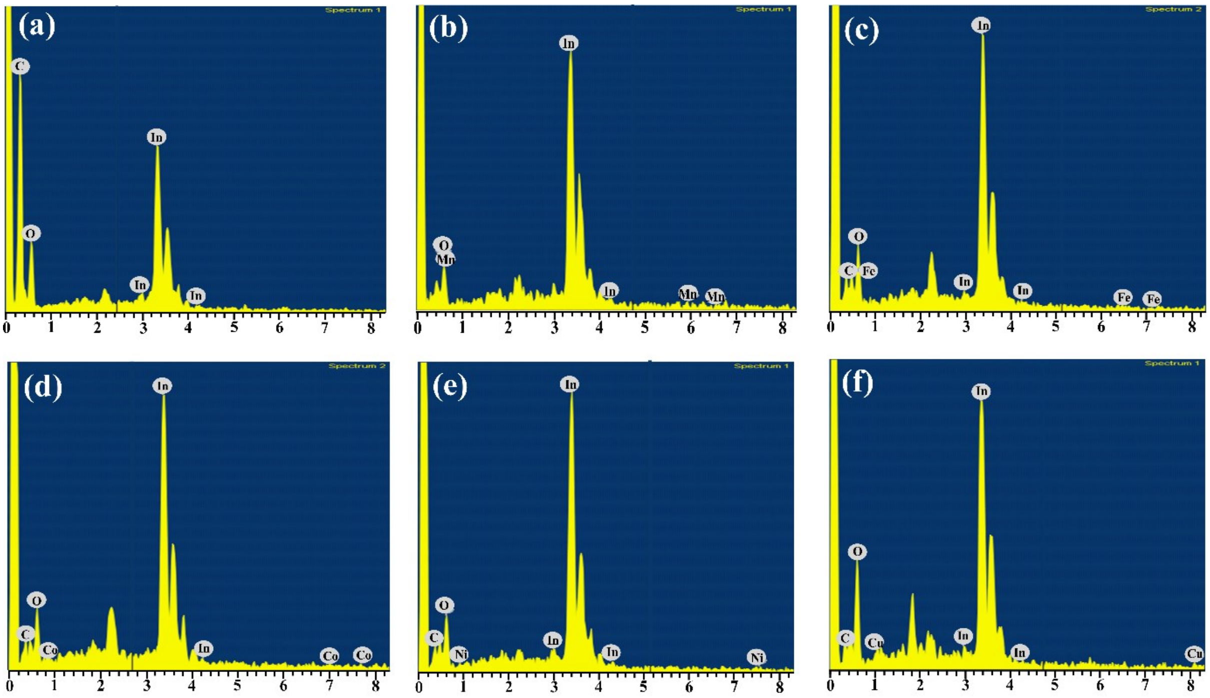

Figure S4. XEDS spectra analysis data from FESEM showing elemental composition of the pure and transition metal ion doped In_2O_3.

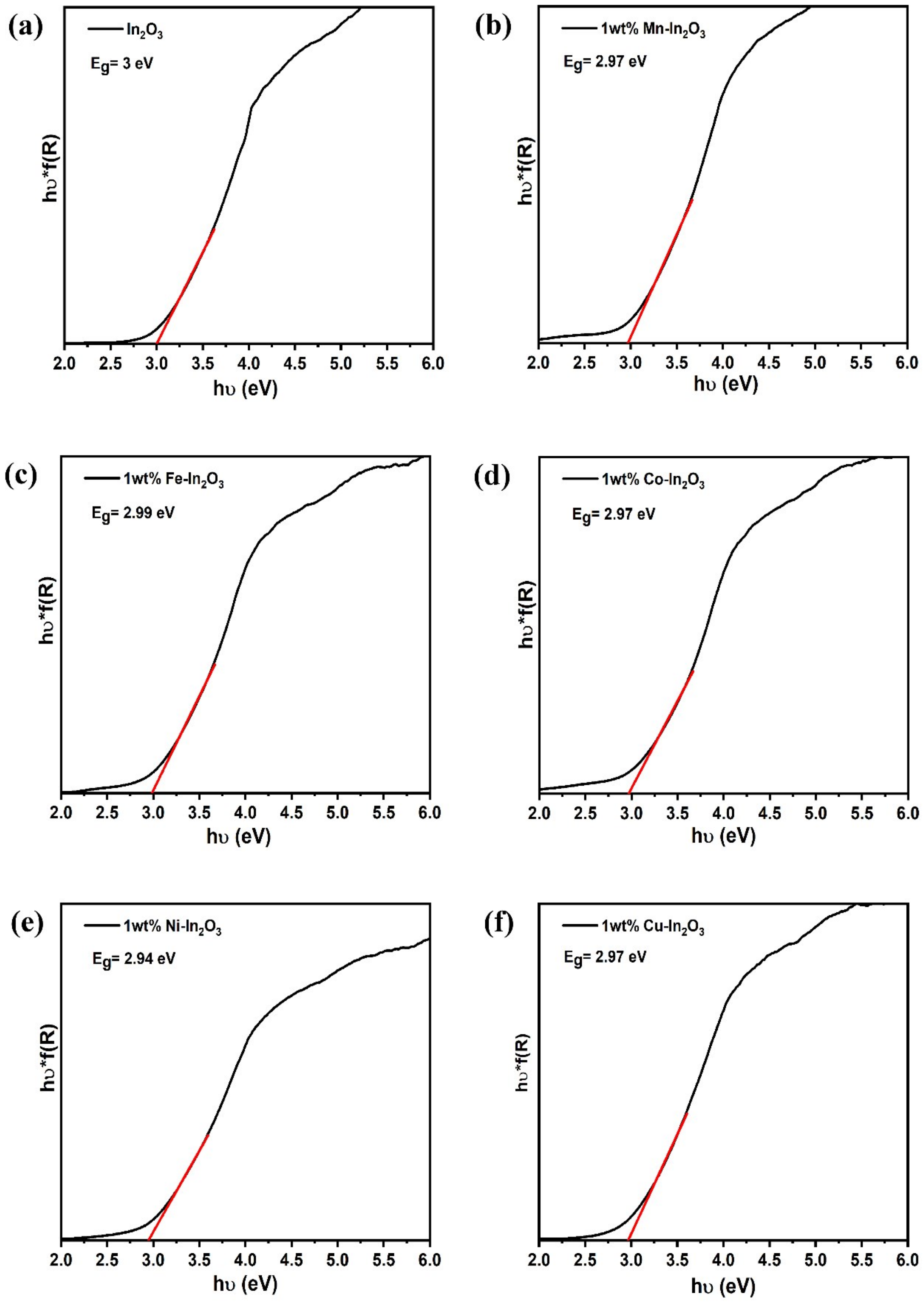

Figure S5. Kubelka-Munk plots for pure and different metal ions doped In_2O_3 nanocubes.

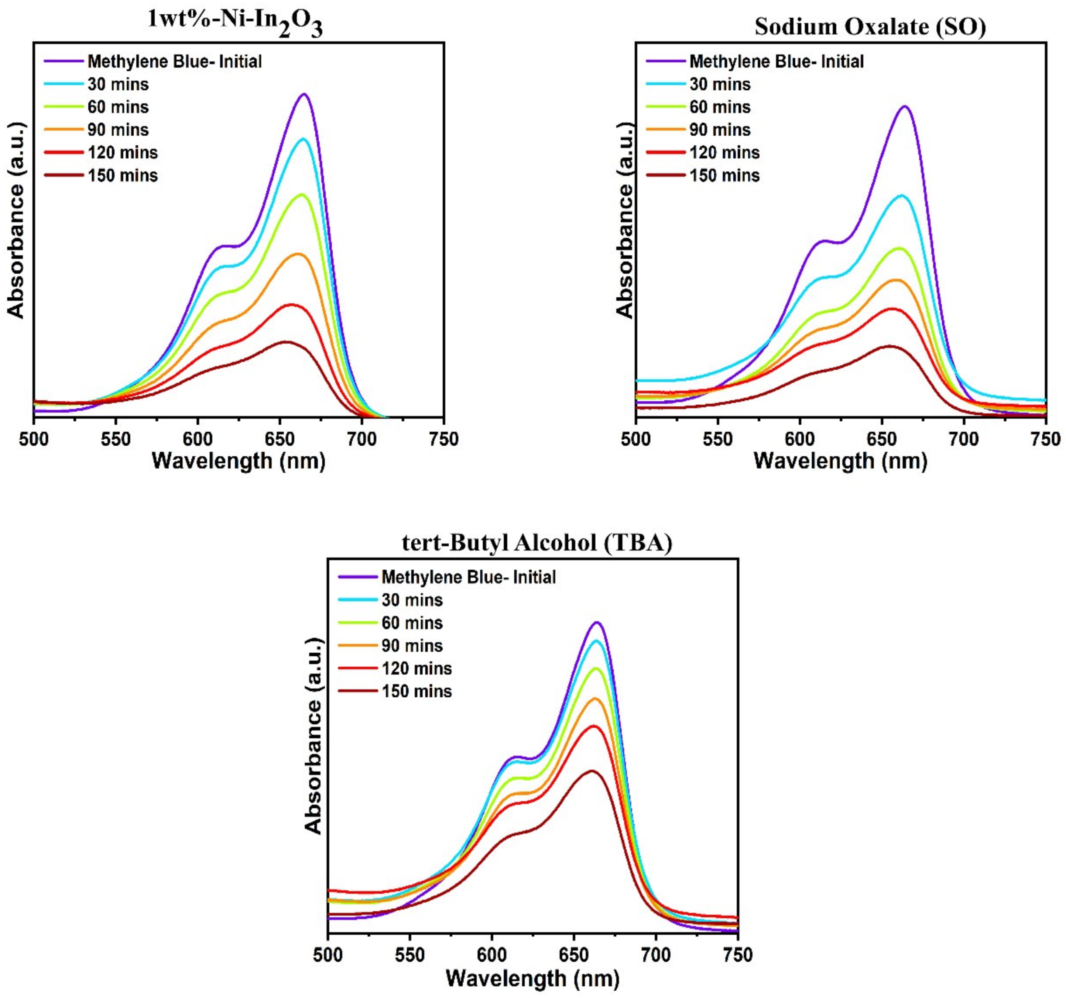

Figure S6. UV-vis absorption spectra of photocatalytic degradation MB under Sunlight irradiation for different time interval by 1wt%Ni-In_2O_3 photocatalyst and also in the presence of different scavengers SO (sodium oxalate) and TBA (tert butyl alcohol) with 1wt%Ni-In_2O_3 photocatalyst.

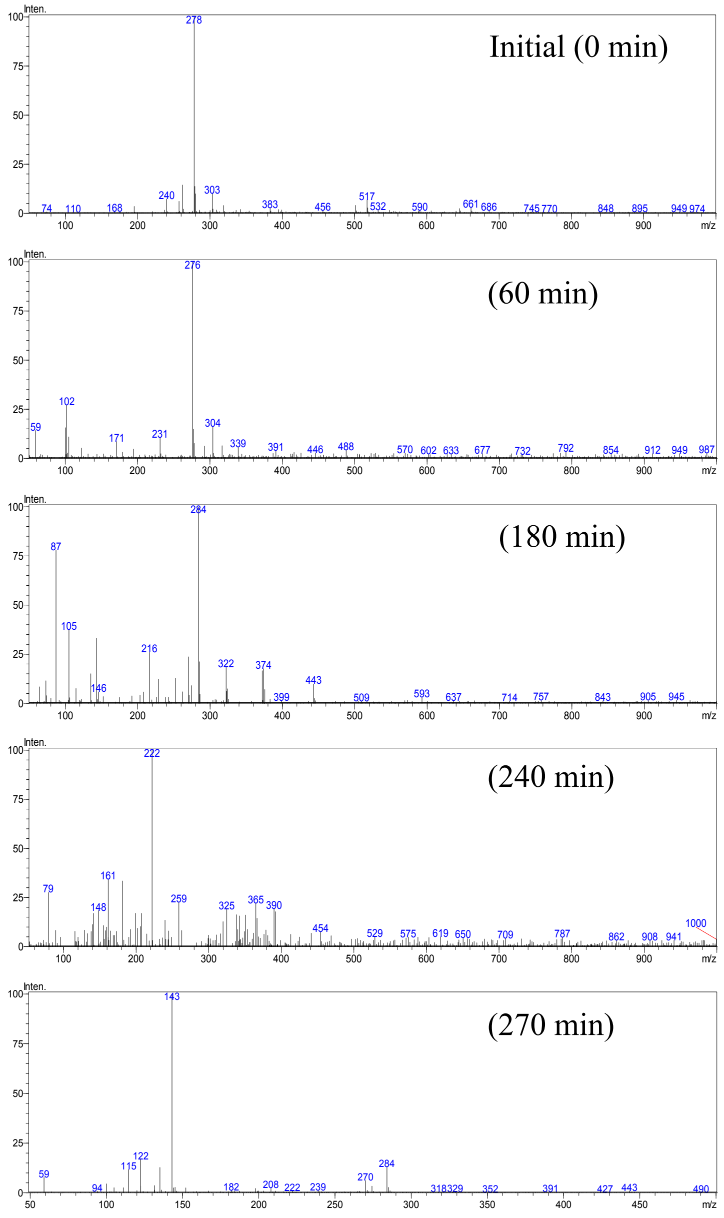

Figure S7. ESI-MS spectra of photodegradation of MB for the time period varied from 0 min to 270 minutes in the presence of 1wt% Ni-In_2O_3 photocatalyst.

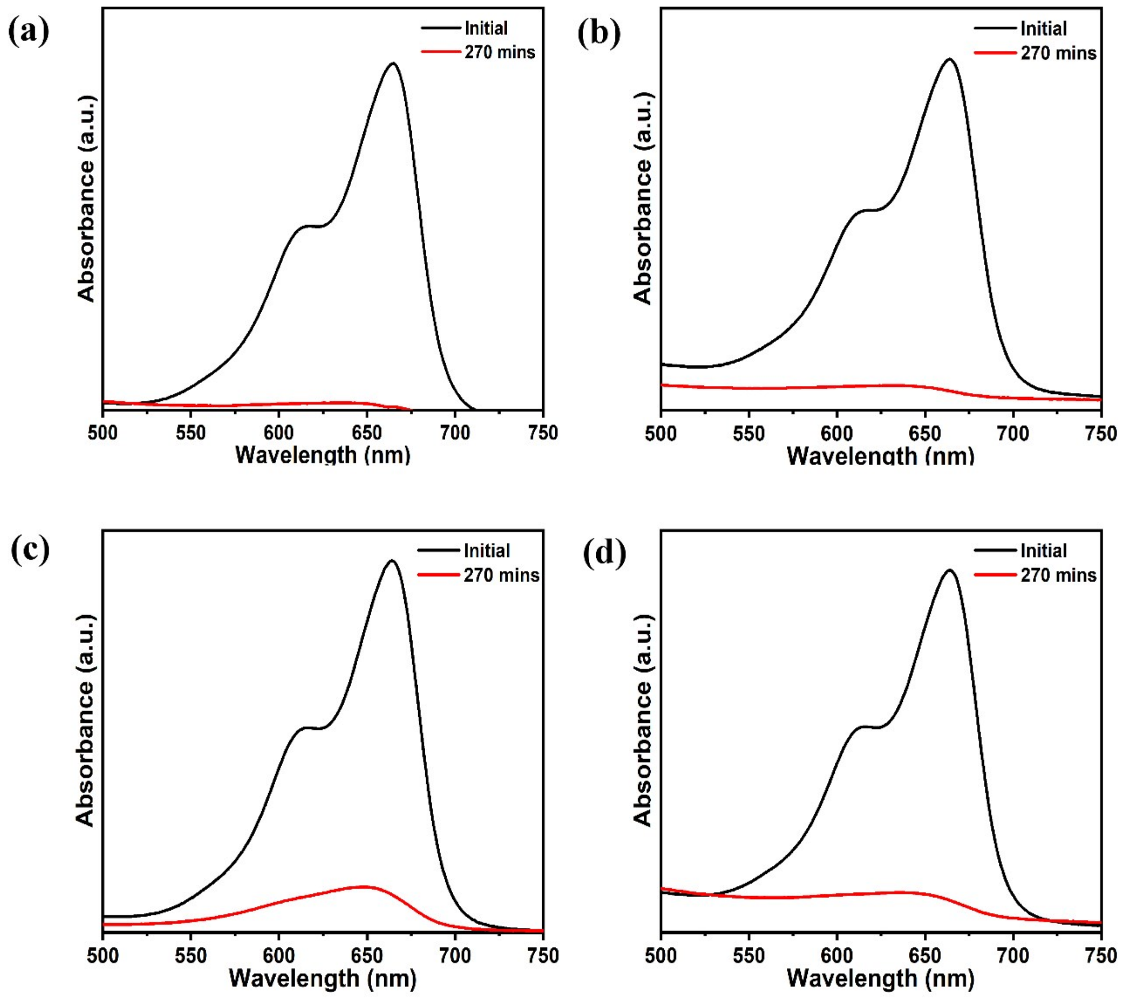

Figure S8. UV-vis absorption spectra of photodegradation of MB before and 270 minutes of visible light irradiation in the presence of 1wt% Ni-In_2O_3 photocatalyst for three different consecutive cycles. (a) 1st cycle, (b) 2nd cycle (c) 3rd cycle and (d) 4th cycle of photocatalysis reaction.

Acknowledgements

This work was financially supported by the DST-SERB (EMR/2016/005795) and UGC-DAE-CSR (CSRKN/CRS-89/2016-17/1130) research projects. The Central Instrumentation Facility of Pondicherry University is gratefully acknowledged.
